# Aurora B functions at the apical surface after specialized cytokinesis during morphogenesis in *C. elegans*

**DOI:** 10.1242/dev.181099

**Published:** 2020-01-08

**Authors:** Xiaofei Bai, Michael Melesse, Christopher G. Sorensen Turpin, Dillon E. Sloan, Chin-Yi Chen, Wen-Cheng Wang, Po-Yi Lee, James R. Simmons, Benjamin Nebenfuehr, Diana Mitchell, Lindsey R. Klebanow, Nicholas Mattson, Eric Betzig, Bi-Chang Chen, Dhanya Cheerambathur, Joshua N. Bembenek

**Affiliations:** 1 Department of Biochemistry, Cellular and Molecular Biology, University of Tennessee, Knoxville, TN 37996, USA; 2 Department of Molecular, Cellular and Developmental Biology, University of Michigan, Ann Arbor, MI 48109, USA; 3 Research Center for Applied Sciences, Academia Sinica, Taipei, Taiwan; 4 Janelia Research Campus, HHMI, Ashburn, VA 20147, USA; 5 Wellcome Centre for Cell Biology, University of Edinburgh, Edinburgh, EH9 3BF, UK

**Keywords:** Apical surface, Aurora B kinase, Cytokinesis, Midbody, Morphogenesis

## Abstract

Although cytokinesis has been intensely studied, the way it is executed during development is not well understood, despite a long-standing appreciation that various aspects of cytokinesis vary across cell and tissue types. To address this, we investigated cytokinesis during the invariant *Caenorhabditis elegans* embryonic divisions and found several parameters that are altered at different stages in a reproducible manner. During early divisions, furrow ingression asymmetry and midbody inheritance is consistent, suggesting specific regulation of these events. During morphogenesis, we found several unexpected alterations to cytokinesis, including apical midbody migration in polarizing epithelial cells of the gut, pharynx and sensory neurons. Aurora B kinase, which is essential for several aspects of cytokinesis, remains apically localized in each of these tissues after internalization of midbody ring components. Aurora B inactivation disrupts cytokinesis and causes defects in apical structures, even if inactivated post-mitotically. Therefore, we demonstrate that cytokinesis is implemented in a specialized way during epithelial polarization and that Aurora B has a role in the formation of the apical surface.

## Abstract (precis)

**Highlighted Article:** Investigations of cytokinesis reveal reproducible patterns during early embryonic development and specialized cytokinesis during epithelial polarization at morphogenesis in *Caenorhabditis elegans*.

## INTRODUCTION

Cytokinesis is the final step of cell division and is not only required to generate two daughter cells, but also regulates development and cellular organization in tissues (Canman et al., 2000; Chen et al., 2013; Herszterg et al., 2014; Li, 2007; Oegema and Hyman, 2006). Signals from the anaphase spindle trigger cleavage furrow ingression (Bringmann and Hyman, 2005; Eggert et al., 2006). After furrowing, cells remain connected at the midbody, a membrane channel containing microtubules, vesicles, a central spindle and contractile ring proteins (El Amine et al., 2013; Green et al., 2012; Hu et al., 2012; Schiel et al., 2013; Skop et al., 2004). The ESCRT machinery mediates the final abscission event (Carlton and Martin-Serrano, 2007; Guizetti et al., 2011; Schiel et al., 2011). Aurora B kinase, part of the chromosome passenger complex, promotes cytokinesis and regulates abscission timing (Carlton et al., 2012; Carmena et al., 2015, 2012; Mathieu et al., 2013; Norden et al., 2006; Steigemann et al., 2009). We sought to investigate the dynamics of cytokinesis during the completely described invariant *Caenorhabditis elegans* embryonic divisions (Sulston et al., 1983).

Cells normally follow the standard cytokinetic process, but several exceptions are known. Some cells do not complete cytokinesis and become polyploid, such as liver and intestinal cells (Amini et al., 2015; Fox and Duronio, 2013; Hedgecock and White, 1985; Lacroix and Maddox, 2012). Germ cells do not complete abscission and remain connected, allowing cytoplasmic exchange (Greenbaum et al., 2007; Haglund et al., 2011; Hime et al., 1996; Maddox et al., 2005), which also occurs in several other cell types (Daniel et al., 2018; McLean and Cooley, 2013; Zenker et al., 2017). The cleavage furrow can be repositioning during anaphase (Ou et al., 2010) or ingress asymmetrically to the apical surface as observed in epithelial tissues (Bourdages et al., 2014; Daniel et al., 2018; Founounou et al., 2013; Guillot and Lecuit, 2013; Herszterg et al., 2014; Higashi and Miller, 2017; Paolini et al., 2015; Wang et al., 2018). Therefore, cytokinesis is altered in different contexts, but more investigation is required to understand the functional purpose of these changes and how they are achieved.

The midbody can be released extracellularly after abscission (Chen et al., 2013; Crowell et al., 2014; König et al., 2017). The midbody remnant (MBR) can persist extracellularly or be engulfed depending on the cell type (Dubreuil et al., 2007; Ettinger et al., 2011; Salzmann et al., 2014). Once internalized, the MBR can elicit intracellular signaling (Peterman et al., 2019). In the early *C. elegans* embryo, MBRs are reproducibly phagocytosed by specific daughter cells (Fazeli et al., 2016; Ou et al., 2014; Singh and Pohl, 2014). This suggests that the MBR regulates cell fate, although the mechanism needs to be further elucidated.

Madin–Darby canine kidney (MDCK) cells can form a lumen, which begins with delivery of apical membrane proteins to the midbody (Li et al., 2014; Reinsch and Karsenti, 1994; Schlüter et al., 2009). Abscission timing and midbody positioning impact lumen formation (Lujan et al., 2016). Vesicle trafficking during cytokinesis promotes lumen formation in other systems (Wang et al., 2014b). Delayed abscission allows vesicles to deliver apical proteins to the membrane in mouse blastomeres (Zenker et al., 2017). The midbody becomes the apical process in chick neuronal progenitors (Wilcock et al., 2007), defines the site of polarization for dendrite extension in *Drosophila* neurons (Pollarolo et al., 2011) and regulates polarity in *Drosophila* neuroblasts (Loyer and Januschke, 2018). Epithelial cells establish new junctions at the midbody to maintain tissue integrity during division (Daniel et al., 2018; Higashi et al., 2016; Wang et al., 2018). The MBR is a polarizing cue during dorsoventral axis formation in the *C. elegans* embryo (Singh and Pohl, 2014; Waddle et al., 1994). The MBR can play a role in cilium formation (Bernabé-Rubio et al., 2016). Further effort is needed to understand how cytokinesis and the midbody regulate pattern formation in tissues.

Forming an apical surface is crucial for proper tissue architecture (Overeem et al., 2015). During morphogenesis in *C. elegans*, cells complete the embryonic divisions and organize into tissues (Chisholm and Hardin, 2005; Leung et al., 1999; Mango, 2007). In epithelial tissues, cells polarize to form apical and basal membranes, reorganize the cytoskeleton and form junctions mediated by adhesion proteins (Bryant and Mostov, 2008; Li and Gundersen, 2008; Niessen et al., 2011). The *C. elegans* intestine polarizes after the E8-E16 division. PAR and adherens junction proteins accumulate in the polar membrane, associate with centrosomes, and then move to the nascent apical midline (Achilleos et al., 2010; Feldman and Priess, 2012). The pharynx divides and polarizes slightly later than the intestine (Portereiko and Mango, 2001). The amphid sensory neurons share features of epithelia (Low et al., 2019) and initially organize into a multicellular rosette with a central apical domain that extends into a dendrite (Fan et al., 2019). Kinetochore proteins regulate microtubules during the initial stages of dendrite extension (Cheerambathur et al., 2019). Cytokinetic regulators have been implicated in epidermal and pharyngeal morphogenesis in roles independent of cell division (Hardin et al., 2008; Portereiko et al., 2004; Von Stetina et al., 2017). Cytokinesis in neuroblasts is important for overlying epidermal cells to migrate during enclosure (Fotopoulos et al., 2013). Therefore, mitotic regulators have mitotic and post-mitotic functions during morphogenesis, although this is not well understood.

We investigated the dynamics of cytokinesis in the invariant *C. elegans* embryonic divisions, including a detailed analysis of several tissues at the onset of morphogenesis. We investigated furrow symmetry, central spindle dynamics, abscission timing and the fate of several different proteins during midbody inheritance. Finally, we investigated mitotic and post-mitotic functions of Aurora B in cytokinesis and at the apical surface in several tissues.

## RESULTS

### Furrow asymmetry and midbody inheritance in the early embryo

**Fig. 1.**
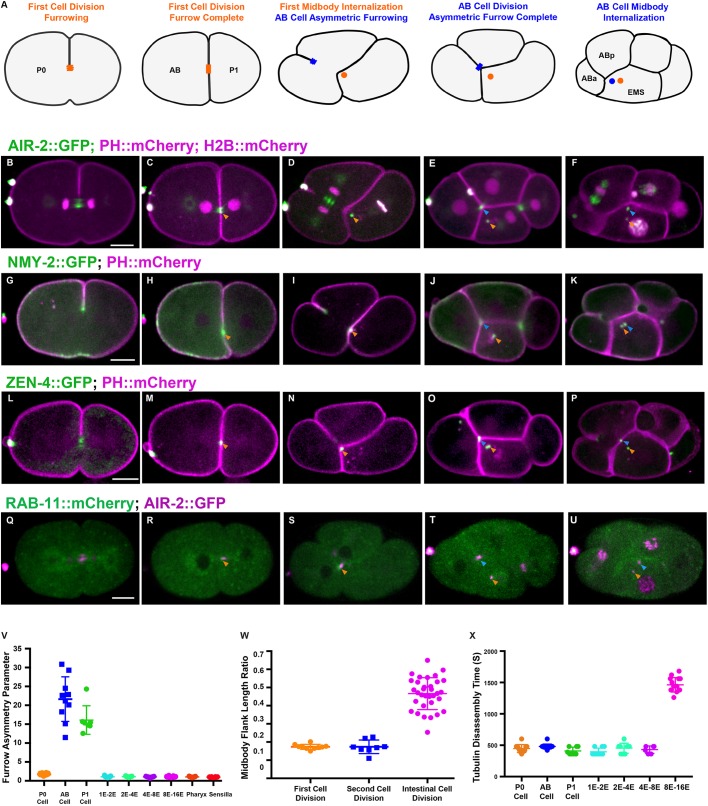
**Cytokinesis in the first two mitotic divisions.** (A) Illustration of cytokinesis in the first two mitotic divisions. The first midbody is shown in orange, the AB midbody is blue. (B-F) Cytokinesis labeled with AIR-2::GFP (green; PH::mCherry and H2B::mCherry in magenta). AIR-2 localizes on the central spindle (B) and the midbody flank (C, orange arrowhead) and the MBR after internalization in AB (D, orange arrowhead). In the AB division, the asymmetric furrow pushes the midzone against EMS (E, blue arrowhead), which engulfs it (F, blue arrowhead). (G-K) NMY-2::GFP (green; PH::mCherry in magenta) localizes to the furrow (G) and midbody ring (H-K). (L-P) ZEN-4::GFP (green; PH::mCherry in magenta) appears on the central spindle (L) and the midbody (M-P). (Q-U) RAB-11::mCherry (green) colocalized with AIR-2::GFP (magenta) at the midbody for a short time before internalization (R-U). Arrowheads in G-U are as described for B-F. (V) Furrow asymmetry parameter is shown for different divisions. (W) Ratio of midbody microtubule length to cell length in different divisions. (X) Quantification of microtubule persistence in different cell divisions. Error bars indicate s.d. Scale bars: 10 μm.

The first mitotic division of P0 generates AB and P1 daughter cells (Fig. 1A). We examined different regulators of cytokinesis to label the central spindle, the cytokinetic furrow and the flank and ring sub-structures of the midbody (Green et al., 2012). To observe the midbody flank region, we imaged Aurora B kinase (AIR-2) (Bembenek et al., 2013), microtubules (Lee et al., 2015), and the membrane trafficking regulator RAB-11 (Sato et al., 2008) (Fig. 1, Fig. S1G-J, Movie 1). We imaged NMY-2 (Nance et al., 2003), which labels the contractile ring and MBR, and the centralspindlin kinesin ZEN-4 (Kaitna et al., 2000), which labels the central spindle and MBR (Fig. 1G-P, Movie 1). The first furrow is slightly asymmetric (Maddox et al., 2007) and the midbody forms in a central position (Fig. 1B,C,G,H,L,M). AIR-2::GFP and tubulin localized to the central spindle and midbody (Fig. 1B,C, Fig. S1G,H, Movie 1). The MBR from the first mitotic division was always inherited by the P1 daughter cell (Fig. 1A,D,I,N,S). Abscission occured within 8 min of furrowing onset, indicated by loss of microtubules (Fig. 1X, Fig. S1H) (Green et al., 2013; König et al., 2017). AIR-2 was lost from the midbody flank but remained on the MBR with other ring components after internalization (Fig. 1D,E,I,J,N,O, Movie 1). Therefore, multiple proteins remain on the MBR after internalization, which may affect its function in P1.

In the second mitotic divisions, several reproducible alterations to cytokinesis were observed. In AB cytokinesis, an asymmetric furrow ingressed from the outer surface until it contacted EMS (Fig. 1D,E,I,J,N,O,S,T). The asymmetry parameter, which is the ratio of furrow ingression distance from each side (Maddox et al., 2007), was 1.7 in the first division, but 21.6 in AB and 16.1 in P1 (Fig. 1V). AIR-2 localized to the spindle midzone in AB, which is swept toward EMS during furrow ingression (Fig. 1E, Fig. S1I,J, Movie 1), and remained associated with the MBR after it was engulfed (Fig. 1D-F, Movie 1). NMY-2 and ZEN-4 also remained on the MBR (Fig. 1I,J,N,O, Movie 1). RAB-11 briefly accumulated at the midbody in the first two mitotic divisions and was not observed on the MBR (Fig. 1Q-U). Therefore, furrow ingression is highly asymmetric during the second division.

The MBR is reproducibly inherited in the early divisions. The AB MBR was invariably engulfed by EMS instead of either of the AB daughter cells (Fig. 1F,K,P,U, Movie 1). Further, the P0 midbody was always inherited by EMS. Abscission timing was relatively fast in both AB and P1 cell divisions as indicated by microtubule disassembly (Fig. 1X, Fig. S1I-J). Disruption of polarity by *par-3(RNAi)* caused random midbody inheritance as previously observed (Ou et al., 2014), and altered furrow symmetry (asymmetry parameter increased to 4.2 in P0, and decreased to 3.0 in AB and 4.4 in P1; Fig. S1K-O). Therefore, both midbody inheritance and furrow symmetry depend on polarity in the early embryo.

### Aurora B remains on the apical surface after E8-E16 gut cytokinesis

**Fig. 2.**
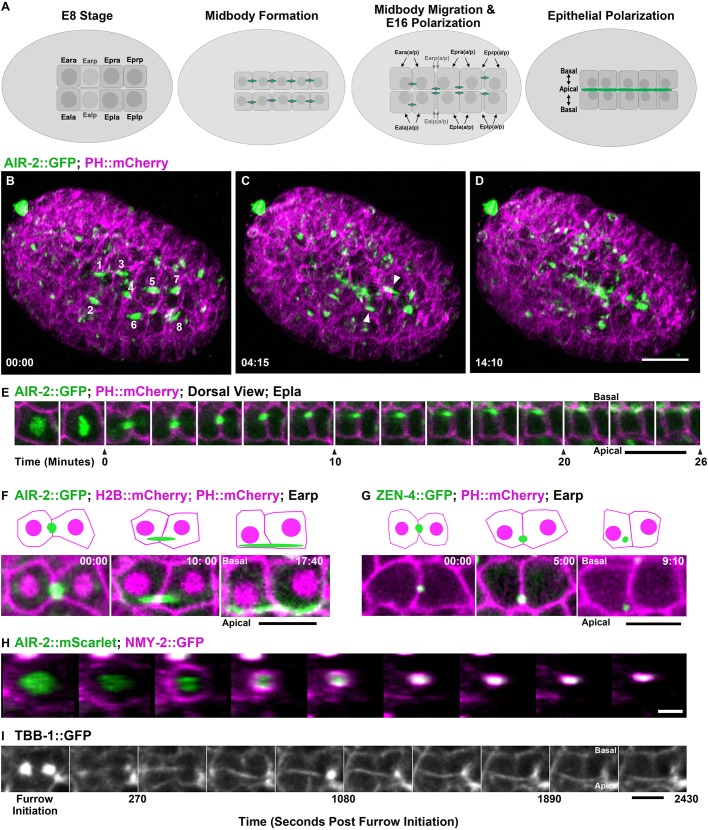
**Midbody migration and Aurora B apical localization after E8-E16 intestinal divisions.** (A) Diagram of intestinal E8-E16 divisions indicating Aurora B localization (green; midbody ring in magenta). (B-D) Lattice light-sheet imaging of E8-E16 divisions with AIR-2::GFP (green) with PH::mCherry (magenta). AIR-2::GFP labels midbodies (labeled 1-8 in B) that migrate to the nascent apical surface (arrowheads, C) where it persists (D). Scale bar: 10 μm. (E) Montage of Epla division with AIR-2::GFP (green; PH::mCherry in magenta) showing midbody formation (t=0) and migration to the apical midline. Scale bar: 5 μm. (F,G) Comparison of AIR-2::GFP (F) and ZEN-4::GFP (G) localization to the apical midline. Scale bars: 5 μm. Time shown in minutes:seconds. In schematics, midbody is green, H2B::mCherry and PH::mCherry are magenta. (H) En face view of the E8-E16 contractile ring labeled with NMY-2::GFP (magenta; AIR-2::mScarlet in green; images taken at 90 second intervals) shows symmetrical furrowing. Scale bar: 2 μm. (I) Single *z*-plane imaging of midbody flank microtubules during Epra cell division. Scale bar: 5 μm.

We next analyzed cytokinesis during morphogenesis, which revealed several novel patterns. The intestine is derived from the E blastomere, which undergoes five embryonic divisions (Leung et al., 1999). The E8 to E16 division occurs around 280 min after the first cleavage and is followed by epithelial polarization (Leung et al., 1999). We observed a highly modified cytokinesis during polarization (Fig. 2A). E8 cells underwent symmetric furrowing over 4.7 min (*n*=15; 1.0 asymmetry parameter; Fig. 1V), to produce a centrally placed midbody (Fig. 2B,H, Fig. S2A,D,G,J, Movies 2-4). Using lattice light-sheet imaging, we observed E8 midbodies migrating to the nascent apical midline over 30 min (Fig. 2C,E, Movie 2). AIR-2::GFP localized on elongated spindle midzone microtubules during movement (Fig. 2E,F, Fig. S2A-C, Movies 2-4). The length of the spindle midzone microtubules relative to the cell was 0.47 (average 4.6 μm/9.8 μm) in the intestinal cell division, which is more than twice that of the early two cell divisions 0.17 (average 9.3 μm/53.4 μm) in P0 and 0.17 (average 7.7 μm/44.3 μm) in AB (Fig. 1W). The midzone microtubules persisted for over 25 min on average before they could no longer be distinguished at the apical midline, indicating a delay in abscission (Figs 1X and 2I). The ring markers ZEN-4 and NMY-2 internalized shortly after the midbody reached the apical midline (553±140 s and 545±179 s, respectively), indicating MBR internalization (Fig. 2G, Fig. S2D-I,O, Movie 3). Therefore, abscission occurs after the midbody migrates to the apical midline. To our knowledge, this is the first observation of apical migration of the midbody in *C. elegans*.

We observed another novel behavior of Aurora B after the E8-E16 division. AIR-2::GFP remained localized at the apical midline (Fig. 2D-F, Fig. S2C,O, Movies 2-4) after ZEN-4 and NMY-2 were internalized (Fig. 2G, Fig. S2D-I,O) colocalizing with the apical polarity marker PAR-6 (Schonegg et al., 2007) (Fig. S2N). Endogenously tagged AIR-2::GFP and immunostained endogenous AIR-2 could also be observed at the apical midline (Fig. S1A,D). The centrosome also migrates to the midline during E16 polarization (Feldman and Priess, 2012; Yang and Feldman, 2015). Centrosomes labeled with γ-tubulin::GFP (Redemann et al., 2010) moved to the apical surface at the same time as AIR-2::GFP localized in the spindle midzone (Fig. S2P). In contrast, the first three E cell divisions exhibited symmetric furrowing (Fig. 1V), rapid abscission timing (Fig. 1X) and no cortical localization of AIR-2::GFP after cytokinesis (Fig. S3). Therefore, after cytokinesis in the E8-E16 divisions, the MBR is internalized but Aurora B remains at the apical surface.

RAB-11 vesicle trafficking of apical proteins to the midbody establishes the apical membrane in other systems (Schlüter et al., 2009). In *C. elegans*, apical RAB-11 endosomes control trafficking in the intestine through adulthood (Sato et al., 2014). We imaged RAB-11::mCherry during the E8-E16 division and found that it colocalizes with AIR-2::GFP, migrates to the apical surface with the midbody and remains localized to the apical surface well after cytokinesis is complete (Fig. S2J-L). Therefore, apical RAB-11 localization is established during E8-E16 cytokinesis and intestinal epithelial polarization.

The anterior and posterior pairs of E16 cells (Ealaa, Earaa, Eplpp and Eprpp) undergo one last embryonic division to achieve the E20 intestine stage. In the four central E8 cells, which do not divide again (Ealp, Earp, Epla and Epra), the midbody migrated to the midline at E8-E16 as described above. However, the midbodies from Eala, Eara, Eplp and Eprp migrated toward the midline at E8-E16, but the AIR-2 signal diminished (Fig. S2M). The midbodies of the E16-E20 divisions also underwent apical migration after symmetrical furrowing (Movie 5). Therefore, apical midbody migration occurs both during and after epithelial polarization in the intestine.

### Inactivation of Aurora B kinase disrupts E8-E16 cytokinesis and proper epithelial polarization

**Fig. 3.**
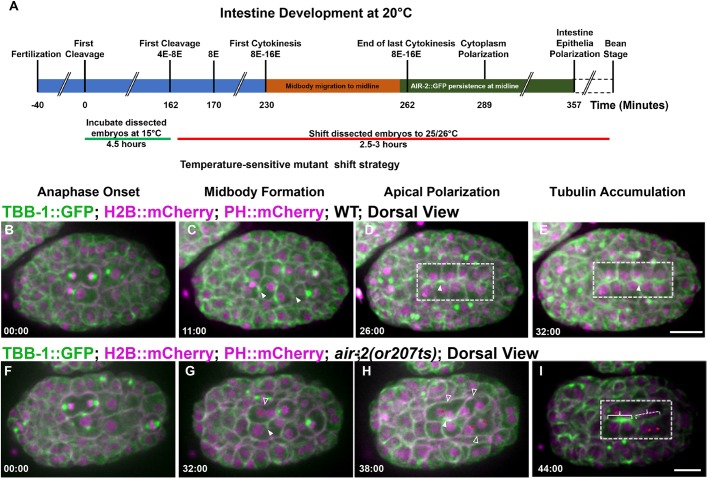
**Aurora B is required for E8-E16 cytokinesis and epithelial polarization.** Microtubule dynamics during the E8-E16 divisions. (A) Time line of embryonic divisions and shifts for temperature-sensitive mutants. (B-E) In wild-type (WT) embryos, spindle midzone microtubules (TBB-1::GFP, green; H2B::mCherry and PH::mCherry shown in magenta) form in late anaphase (arrowheads, C) and migrate to the apical midline (D) and persist (E). (F-I) In *air-2(or207)* embryos, spindle midzone microtubules are diminished (G, filled arrowhead). When cytokinesis failures occur (G,H, unfilled arrowheads), nuclei fail to reach the apical midline (G-I, red asterisks) and apical microtubule accumulation is reduced (dashed bracket indicates failed divisions, solid bracket indicates successful divisions with apical accumulation). Dashed boxes indicate gut cells. Scale bars: 10 μm.

We next investigated the function of Aurora B and other cytokinetic regulators during the E8-E16 division. To bypass the essential function of cytokinetic regulators during the early divisions, we inactivated temperature-sensitive (ts) mutants after isolating two-cell embryos and incubating them at the permissive temperature (15°C) until different stages before shifting them to the non-permissive temperature (26°C) until they hatched (Fig. 3A). After epidermal enclosure, the embryo begins to elongate during the bean stage, followed by the comma, 1.5-fold, 2-fold and 3-fold stages, after which the larvae hatches from the egg. Two-cell *air-2(or207)* (Severson et al., 2000) embryos had only 53.6% (37/69) viability when kept at 15°C, indicating that this mutant is sick even at permissive temperature, whereas wild-type N2, *zen-4(or153)* (Severson et al., 2000) and *spd-1(oj5)* (O'Connell et al., 1998) embryos were 100% viable at 15°C (Table S1). Embryos shifted from 15°C to 26°C after 4.5 h (corresponding to late E4 to early E8 stages) or 6.5 h (corresponding to E8-E16 stage or early bean stage) showed significant lethality in both *air-2(or207)* and *zen-4(or153)*, but not *spd-1(oj5)* (Table S1). Mutant embryos shifted after most divisions had finished at the comma to 1.5-fold stage hatched at a rate similar to those at permissive temperature (Table S1). The *air-2(or207)* mutant has penetrant cytokinesis failure immediately after inactivation in early embryos (Severson et al., 2000) but this was not observed in in older embryos (37.8% cytokinesis failure in *n*=138 divisions; Fig. S5B,D; see Materials and Methods). Consistent with this, only mild lagging chromosome segregation defects were observed in older *air-2(or207)* mutants (Fig. S5F). A temperature-sensitive mutant of the INCENP homolog *icp-1(or663ts)* (Davies et al., 2014) also did not have penetrant cytokinesis failures at the E8-E16 division (9%, *n*=22 divisions). Therefore, these mutants allow us to bypass early divisions but do not yield rapid-onset, highly penetrant cell division phenotypes later in development.

Next, we tested whether AIR-2 was required for the specialized E8-E16 divisions and epithelial polarization. We first asked whether microtubule organization required AIR-2. *air-2(or207)* mutant embryos shifted at the E4-E8 stage had reduced spindle midzone microtubules relative to wild type (Fig. 3B-I, Movie 6). Inactivation of *air-2(or207)* caused cytokinesis failure in 27% of the observed E8 cells and a failure to normally polarize all nuclei at the midline (Fig. 3F-I, Fig. S5A-C). In *air-2(or207)* E8 cells that did not fail cytokinesis, weak spindle midzone microtubules moved to the apical surface where microtubules accumulated (Fig. 3F-I, Movie 6). In neighboring cells that failed cytokinesis, microtubule accumulation at the apical midline was diminished, which was most obvious when both left and right E8 divisions failed at the same time (Fig. 3H, Fig. S5A-C, Movie 6). Therefore, AIR-2 regulates central spindle microtubules and completion of cytokinesis during the E8-E16 divisions. Furthermore, nuclear polarization and apical microtubule accumulation are disrupted when cytokinesis fails after inactivation of Aurora B.

**Fig. 4.**
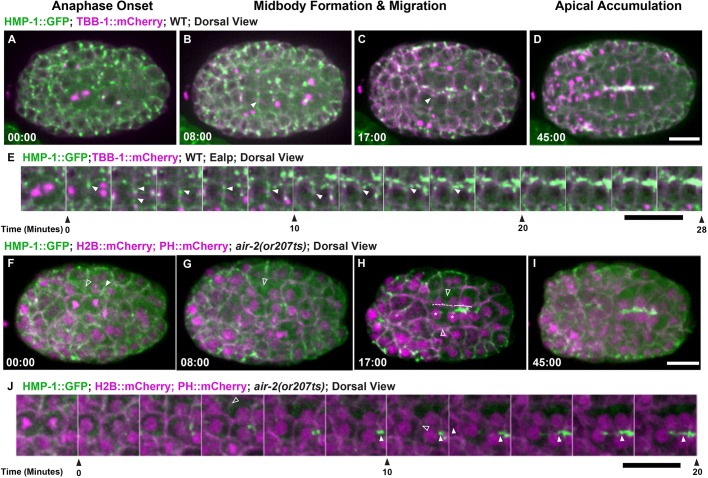
**Aurora B is required for adhesion dynamics during E8-E16 cytokinesis.** Adhesion dynamics during E8-E16 division and polarization. (A-D) HMP-1::GFP (green; microtubules in magenta) localizes to the furrow and midbody (B, arrowhead) during cytokinesis. HMP-1::GFP migrates with the midbody (C, arrowhead) to the apical surface where it accumulates after polarization (D). (E) Montage of HMP-1::GFP during E8-E16 division shows furrow and midbody (arrowheads) migration to the apical midline. (F-I) Aurora B mutants have reduced HMP-1::GFP on the furrow and midbody (F, unfilled arrowheads). When cells fail cytokinesis (G,H, unfilled arrowheads), HMP-1 accumulation is delayed (dashed bracket shows failed cytokinesis, solid bracket indicates successful E8 division with apical accumulation). Asterisks in H indicate nuclei. (I) HMP-1 signal eventually spreads along the midline. (J) Montage of HMP-1::GFP in Aurora B mutant E8-E16 cells that fail cytokinesis (unfilled arrowheads indicate furrow regression) and have delayed apical accumulation. Filled arrowheads indicate HMP-1 signal. Scale bars: 10 μm.

The adhesion complex accumulates at the apical surface during polarization to promote gut lumen formation after the E8-E16 divisions (Achilleos et al., 2010). We imaged the α-catenin HMP-1::GFP (Marston et al., 2016), part of the cadherin-catenin adhesion complex that links cell-cell contacts with actin. PAR and adhesion complexes localize to cortical foci (Achilleos et al., 2010) and move with centrosomes to the apical midline (Feldman and Priess, 2012). We also observed HMP-1::GFP at the furrow and midbody throughout apical migration (Fig. 4A-E, Movie 7). HMP-1::GFP localized to the furrow and membrane adjacent to the midbody in the first three E cell divisions (Fig. S4), indicating that this localization is not specific to the E8-E16 division. This dynamic adhesion localization during cytokinesis may be important to maintain proper cell contacts during the disruptive process of division in the early embryo. In *air-2(or207)* mutants, furrow HMP-1::GFP was reduced during E8 cytokinesis (Fig. 4F-J, Fig. S5D,E, Movie 7). In E8 *air-2(or207)* divisions that completed cytokinesis, HMP-1::GFP signal accumulated at the apical midline (Fig. 4I, Fig. S5D,E, Movie 7). However, in *air-2(or207)* E8 divisions that failed cytokinesis, accumulation of HMP-1::GFP was delayed especially when pairs of E8 daughters on opposite sides of the midline both failed (Fig. 4F-J, Fig. S5D,E, Movie 7). Therefore, Aurora B inactivation leads to reduced furrow localization of α-catenin and disrupts cytokinesis, delaying accumulation of α-catenin at the apical surface during polarization.

**Fig. 5.**
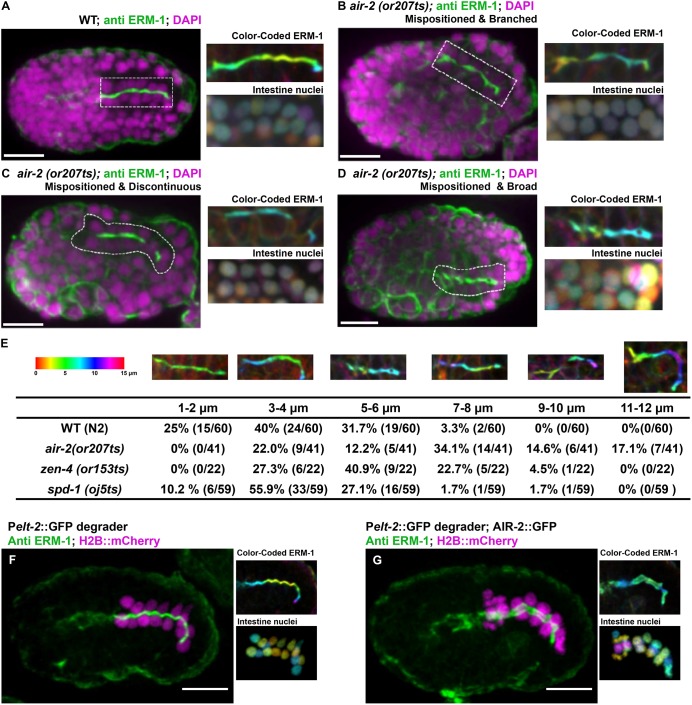
**Gut morphogenesis is disrupted in cytokinesis mutants.** Apical surface staining after E8-E16 division and polarization. (A) ERM-1 apical staining (dashed rectangle) in wild-type (WT) bean-stage embryos. Maximum *z*-projected images of ERM-1 and nuclei color-coded according to *z*-depth (scale shown in F) show tissue organization. (B-D) In *air-2(or207)* embryos, apical surfaces are mispositioned (B-D), branched (B), contain gaps (C) or have broader staining (D). (E) Quantification of the defective apical *z*-plane distribution in different mutants (more colors indicate greater distortion in the *z*-plane). (F) ERM-1 staining and distribution of nuclei in a control embryo (GFP degrader only expressed) shows normal lumen width (1.15±0.11 µm, *n*=10) and nuclear distribution. (G) Endogenous AIR-2::GFP degradation in the intestine results in significantly broadened ERM-1 staining (2.53±0.33 µm, *n*=8) and disorganized nuclei. Scale bars: 10 μm.

In order to understand the effect of cytokinesis failure on lumen formation, we performed staining of the polarized gut using apical markers. We shifted mutant E4-E8 embryos to 26°C and fixed after intestinal polarization. We evaluated the apical surface by staining for the Ezrin-Radixin-Moesin homolog ERM-1 (van Furden et al., 2004). We stained *air-2(or207)*, *zen-4(or153)*, *spd-1(oj5)* and *icp-1(or663)* and frequently observed deformed and binucleate cells in *air-2(or207*) and *zen-4(or153)* but not *spd-1(oj5)* mutants (Table 1, Fig. 5, Fig. S6). In all cases, ERM-1 was localized to the apical surface of the intestine and pharynx (Fig. 5A). However, ERM-1 staining was broadened, branched and/or discontinuous in *air-2(or207)* embryos (Fig. 5B-E, Table 1). Comma-stage *air-2(or207)* embryos still had disrupted ERM-1 staining, indicating that these defects are not resolved later in development (Fig. S6B,E). Furthermore, the intestine was highly mispositioned within the embryo (Fig. 5E) and the nuclei were disorganized (Fig. 5A-D, insets). Other apical markers also localized to the disorganized apical surface, including PAR-3, DLG-1 and IFB-2 (Fig. S6F-K). *zen-4(or153)* embryos had penetrant branched and discontinuous apical ERM-1 staining that was mispositioned at a lower rate (Table 1, Fig. S6C,E). *spd-1(oj5)* embryos displayed a significant but lower rate and severity of lumen defects (Table 1, Fig. S6D,E) despite having no lethality (Table S1) and minimal cytokinesis failures. We observed AIR-2::GFP dynamics in *spd-1(oj5)* E8-E16 divisions and found that AIR-2::GFP was lost from spindle midzone microtubules and instead accumulated at spindle poles, which moved to the apical surface in *spd-1(oj5)* embryos (Fig. S6E). Daughter cell pairs did not remain together in *spd-1(oj5)* embryos, indicating that spindle midzone facilitates polarization (Fig. S6E). Therefore, AIR-2::GFP can still reach the apical surface through a compensatory mechanism when SPD-1 is inactivated. This compensatory mechanism is not perfect, leading to significant but reduced lumen defects. Finally, *icp-1(or663ts)* mutant embryos also had abnormal ERM-1 staining (Fig. S6L-M). Therefore, we conclude that Aurora B, the spindle midzone and other regulators of cytokinesis are required for normal apical lumen formation in the gut.

**Table 1.**
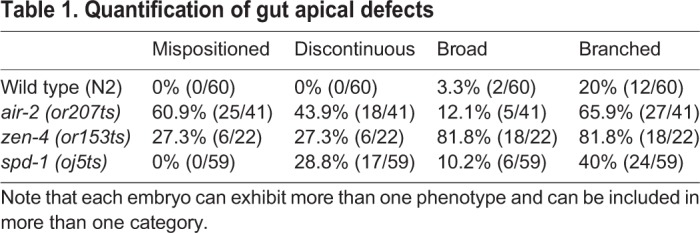
**Quantification of gut apical defects**

To inactivate AIR-2 more precisely, we tested whether tissue-specific depletion in the gut would cause lumen defects. We used a GFP-degradation system to deplete endogenously GFP-tagged Aurora B starting during the E8 stage (Wang et al., 2017). For comparison, we also depleted endogenously tagged NMY-2::GFP and ZEN-4::GFP (Dickinson et al., 2013; Lee et al., 2018). We quantified binucleate intestinal cells in L1 animals and observed 53.7% in AIR-2-depleted (*n*=23 animals), 19.7% in ZEN-4-depleted (*n*=10), 13.6% in NMY-2-depleted (*n*=7) and 3% in control (*n*=10) embryos, indicating significant rates of cytokinesis failure. We stained embryos with ERM-1 after gut polarization to observe lumen defects. We measured four points along the length of the gut lumen and obtained an average width. In control embryos, the apical lumen was 1.15±0.11 µm wide (*n*=10); AIR-2::GFP depletion caused lumens to be twice as wide on average (2.53±0.33 µm, *n*=8, *P*<3.8×10^−7^; Fig. 5F,G). The widest part of the lumen after AIR-2::GFP depletion was 2.94±0.41 µm (*P*<3.33×10^−6^), but only 1.27±0.03 µm in control embryos. Depletion of NMY-2::GFP (1.05±0.17 µm, *n*=7) or ZEN-4::GFP (0.99±0.11 µm, *n*=4) did not cause wide lumens, despite having cytokinesis failures (Fig. S6M,N). Therefore, tissue-specific depletion of Aurora B but not other cytokinetic regulators in the E8 gut leads to a highly consistent defect in the width of the lumen, consistent with a function for Aurora B at the apical surface.

### Apical midbody migration and AIR-2 apical localization in the pharynx

**Fig. 6.**
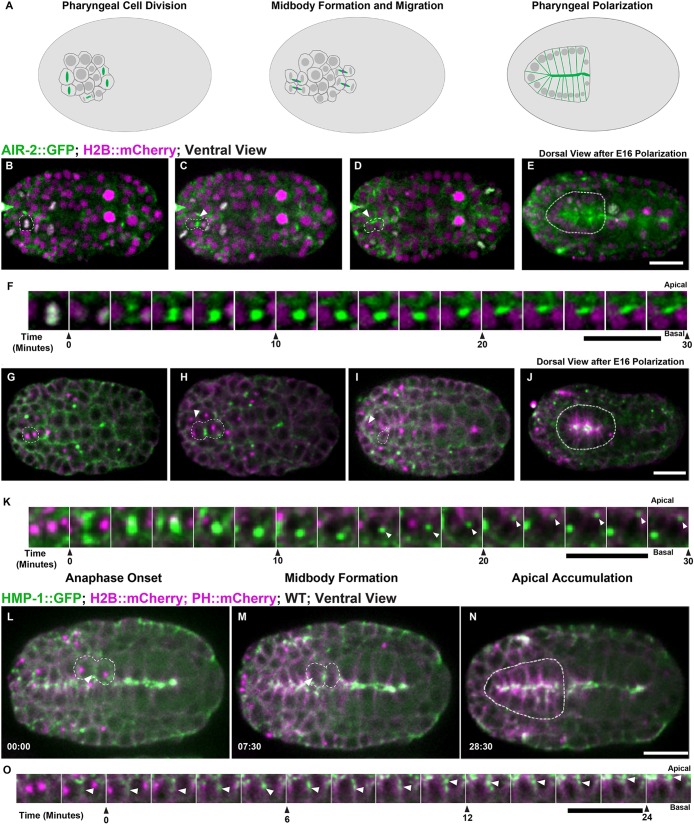
**Cytokinesis during pharyngeal precursor cell polarization.** (A) Illustration of cell division in PPCs with Aurora B (green; midbody ring in magenta). (B-E) PPC division labeled with AIR-2::GFP (green; H2B::mCherry in magenta) from both ventral (B-D, dashed line highlights one cell, arrowhead indicates midbody) and dorsal (E, dashed line highlights pharynx) views. AIR-2::GFP localizes to chromosomes in metaphase (B), moves to the central spindle in anaphase (C), and appears on the midbody which moves toward the midline (D). AIR-2 persists at the pharyngeal apical surface for an extended time (E). (F) Montage showing AIR-2::GFP migrating toward the midline. (G-K) Imaging of NMY-2::GFP (green; TBB-1::mCherry in magenta), during midbody migration to the midline (I,K). NMY-2::GFP accumulates at the midline during apical constriction (J). (L-N) During PPC cytokinesis, α-catenin (HMP-1::GFP, green; tubulin in magenta) accumulates on the furrow (arrowhead in L) and adjacent to the midbody (arrowhead, M) before accumulating at the midline (N). (O) Montage of HMP-1::GFP in PPC cell at the furrow, midbody and apical midline. Time shown in minutes:seconds. WT, wild type. Scale bars: 10 μm.

We also observed cytokinesis during the terminal divisions in the pharynx. The pharynx forms from more than 80 pharyngeal precursor cells (PPCs) and the final divisions occur 310-325 min after the first cleavage (Sulston et al., 1983). PPCs polarize and undergo apical constriction to form a lumen by 355 min (Rasmussen et al., 2013, 2012). We imaged from both dorsal and ventral aspects to observe PPC divisions and apical polarization (Fig. 6, Movie 8). We also used lattice light-sheet microscopy, which provides higher spatial resolution during the pharyngeal cell division (Movie 9). PPCs underwent symmetric furrowing that yielded a centrally placed midbody between daughter cells (Fig. 6A,F, Movie 8). PPC midbodies migrated from their central position between daughter cells toward the apical midline of the forming pharyngeal bulb (Fig. 6F,K,O, Movie 9). In PPC terminal divisions, AIR-2::GFP appeared on the spindle midzone, migrated with the midbody to the apical midline and persisted there (Fig. 6B-F, Movie 9). We confirmed apical localization using endogenously tagged AIR-2::GFP and immunofluorescence against endogenous AIR-2 (Fig. S1B,E). ZEN-4::GFP appeared on midbodies, migrated toward the apical surface, and rapidly disappeared (Fig. S7A-E, Movie 8). NMY-2::GFP also labeled midbodies and moved to the apical surface, but remained there during apical constriction (Fig. 6G-K) (Rasmussen et al., 2012). RAB-11 and tubulin accumulated and remained localized to the apical surface after polarization (Fig. S7F,G). AIR-2 partially colocalized with PAR-6 and γ-tubulin::GFP (Toya et al., 2010) at the apical membrane (Fig. S7H,I). HMP-1::GFP localized to the furrow and midbody as it migrated to the apical midline where it accumulated after polarization (Fig. 6L-O). Staining of embryos shifted slightly later in development revealed severe defects in pharyngeal formation in *air-2(or207)*, *zen-4(or153)* and *icp-1(or663ts)* mutant embryos (Fig. S6B-D,L). Therefore, similar patterns of symmetric furrowing, midbody migration and apical localization of AIR-2 are observed during epithelial polarization in the intestine and pharynx in *C. elegans*.

### Apical midbody clustering and AIR-2 dendrite localization in sensilla neurons

**Fig. 7.**
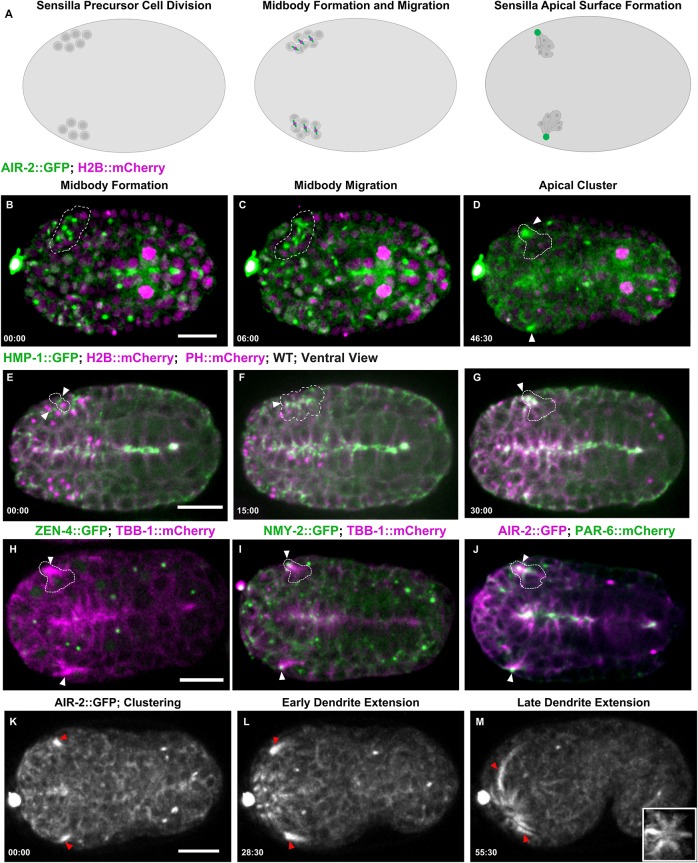
**Midbody components label dendrites of sensilla neurons.** (A) Diagram of SPC divisions with Aurora B (green; midbody ring in magenta). (B-D) Cytokinesis in SPCs expressing AIR-2::GFP (green; H2B::Cherry in magenta) gives rise to multiple midbodies (dashed outline, B,C) that cluster together (arrowheads, D). (E-G) HMP-1::GFP accumulates at the furrow and midbody (arrowheads, E,F), accumulates at the apical cluster, and remains at the tip (G) during dendrite extension. (H) ZEN-4::GFP (green; microtubules in magenta) is internalized and degraded before the microtubule-rich cluster forms (arrowheads). (I) NMY-2::GFP (green; microtubules in magenta) remains at the tip of the dendrite as it extends (arrowheads). (J) PAR-6::mCherry (green) and AIR-2::GFP (magenta) colocalizes to the cluster (arrowheads), indicating that this is the apical surface. (K-M) AIR-2::GFP labels the dendrites during extension. Inset in M is a rotated maximum *z*-projection of sensilla after dendrite extension. Time shown in minutes:seconds. Scale bars: 10 μm.

Finally, we observed a unique form of cytokinesis in sensory neuron precursor divisions. The *C. elegans* amphid sensilla contain 12 neurons with dendrites that extend processes into the tip of the mouth. During morphogenesis, amphid neurons anchor at the tip of the animal and migrate back to extend dendrites (Heiman and Shaham, 2009). There are ten precursor cell divisions that occur between 280 and 400 min after the first cleavage to form the sensilla neurons (Sulston et al., 1983). Our observations show cytokinesis occurs in these sensilla precursor cells (SPCs) just before dendrite morphogenesis (Fig. 7A). SPCs underwent symmetrical furrowing with midbodies forming centrally between the daughter cells (Fig. 7B, Movies 10, 11). A group of at least six daughter cell pairs divided, initially forming multiple midbodies as observed with both confocal and lattice light-sheet imaging (Fig. 7C, Movies 10, 11). These midbodies migrated into a central cluster over a 60-min time window (Fig. 7D). AIR-2, RAB-11 and tubulin persisted in these clusters (Fig. 7D, Fig. S8A,B), whereas ZEN-4 rapidly disappeared, suggesting that abscission and MBR internalization had occurred (Fig. 7H, Movie 10). Cluster staining was observed with AIR-2 immunofluorescence or endogenous AIR-2::GFP (Fig. S1C,F). NMY-2::GFP migrated with the midbody to the cluster and persisted at the tip of the dendrites during extension (Fig. 7I, Movie 10). PAR-6 localized to this cluster, indicating that this site is the apical surface of these cells, which also accumulates γ-tubulin::GFP (Fig. 7J, Fig. S7C,D). Recently, it was found that SPCs form a multicellular rosette with PAR-6 at the center, indicating that the midbody moves to the apical surface at the center of this rosette structure (Fan et al., 2019). HMP-1::GFP was observed at the furrow and midbody and migrated to the apical cluster and remained there during dendrite extension (Fig. 7E-G). Therefore, the midbody moves to the apical surface of the sensilla rosette where Aurora B and other midbody components accumulate.

After formation of the apical rosette, we observed that this region extends anteriorly toward the tip of the animal. AIR-2 and tubulin remained localized along the dendritic extension during elongation (Fig. 7K-M, Fig. S8D,E, Movie 12). As dendrites extended, other AIR-2 foci formed within the anterior region of the embryo and migrated toward the tip until six sensilla appeared (Fig. 7M, inset, Movies 12, 13). Although the individual cell divisions could not be easily discerned, these data suggest that other sensilla in the tip of the animal form through a similar process. Neuronal cell polarization may share mechanisms with epithelial morphogenesis (Low et al., 2019; McLachlan and Heiman, 2013), suggesting that modified cytokinesis may regulate epithelial polarization. Therefore, the midbody migrates from its original position at the end of furrowing to the apical surface in several tissues during morphogenesis. Interestingly, Aurora B remains localized at the apical surface well after completion of cytokinesis.

### Aurora B has a post-mitotic function in dendrite formation

**Fig. 8.**
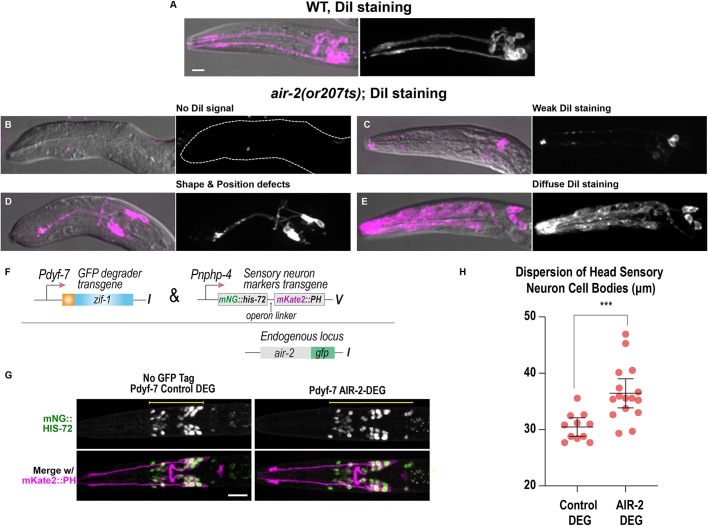
**Cytokinesis mutants have disrupted sensilla neuron morphology.** (A-E) Dendrite and neuron morphology revealed by DiI staining in L1 larvae. (A) In wild type (WT), two dendrite bundles and amphid and phasmid neurons are labeled. (B-E) *air-2(or207ts)* mutants show no DiI signal (B), weak signal (C), dendrite shape and positioning defects (D) and diffuse staining throughout the head of the animal (E). Dashed line outlines indicate animal position in B. (F) Construct used for post-mitotic degradation of AIR-2::GFP in sensory neurons. (G) Sensory neuron nuclei (green) and plasma membranes (magenta) for the indicated conditions. Scale bars: 10 μm. (H) Quantification of sensory neuron cell body distribution, measured as indicated by the yellow lines in G. Error bars are the 95% confidence interval. ***P<0.001 (two-tailed unpaired *t*-tests in GraphPad Prism; *n*=12 for control DEG and *n*=16 for AIR-2 DEG).

We tested whether Aurora B kinase and other cytokinesis components were required for sensilla formation. Cilia that form at the end of sensilla dendrites are exposed to the environment and can take up lipophilic dyes such as DiI (Hedgecock and White, 1985; Perkins et al., 1986). We inactivated cytokinesis mutants at different embryo stages and stained surviving L1 larvae with DiI. In wild type, amphid neurons were clearly labeled by DiI (Fig. 8A). In *air-2(or207)* mutants, we observed numerous defects in neuronal staining (Fig. 8B-E), including no DiI staining, indicating that none of the sensilla reached the environment (Table S2). *zen-4(or153)* larvae showed severe DiI staining defects, which was dramatically reduced if embryos were shifted after the final divisions at the comma to 1.5-fold stage (Fig. S9A-C, Table S2). *spd-1(oj5)* animals had weak defects revealed by DiI staining but never showed a complete lack of staining (Fig. S9D, Table S2). Therefore, several cytokinetic regulators, including AIR-2, are required for dendrite formation.

Finally, we tested whether tissue-specific, post-mitotic depletion of AIR-2 would disrupt sensilla formation. We depleted endogenously tagged AIR-2::GFP from the sensory neurons by expressing the GFP degrader under the *dyf-7* promoter (Fig. 8F), which is activated after the SPC divisions (Cheerambathur et al., 2019). AIR-2::GFP depletion in SPCs resulted in a wider distribution of cell bodies in L1 animals compared with control animals (Fig. 8G,H). This phenotype is also caused by the depletion of other kinetochore proteins, which also localize to the dendrite extension. Post-mitotic depletion of kinetochore proteins causes defects in the dendrite extension process, disrupts microtubule dynamics and also causes dispersion of the neuronal cell bodies (Cheerambathur et al., 2019). These data provide strong evidence that AIR-2 functions at the apical domain of the extending dendrite and has a role independent of its function during cytokinesis. Therefore, AIR-2 function is required for specialized cytokinesis during epithelial polarization and the subsequent formation of the apical surface during morphogenesis.

## DISCUSSION

Our results reveal complex and reproducible patterns of cytokinesis during the invariant embryonic divisions in *C. elegans*. We observed reproducible alterations to furrow symmetry, central spindle length, abscission timing, midbody movement and MBR inheritance. We show that cells are completing cytokinesis when they polarize during morphogenesis. Aurora B inactivation disrupts cytokinesis and epithelial polarization. Many cells in the lineage divide and produce an apoptotic daughter cell rather than finish the divisions early, which may be due to the role of cytokinesis in polarization. Indeed, modified cytokinesis in the Q neuroblast generates a smaller daughter cell that undergoes apoptosis, which is prevented as cytokinesis parameters change (Ou et al., 2010). Cytokinesis is the transition into interphase and an ideal time to reorganize cellular architecture. Investigating the developmental plasticity of cytokinesis will be a fascinating question for future studies.

Asymmetric furrows drive efficient furrowing and help maintain proper cell contacts during cytokinesis (Founounou et al., 2013; Guillot and Lecuit, 2013; Maddox et al., 2007; Morais-de-Sá and Sunkel, 2013). Asymmetry of furrow ingression may contribute to the differential sensitivity to actin regulation in the four-cell embryo (Davies et al., 2018). The asymmetric furrow may facilitate MBR inheritance by EMS and it is worth noting that MS accumulates four MBRs, which could regulate its fate (Singh and Pohl, 2014). In polarized epithelial divisions, the furrow constricts to the apical surface to position the midbody (Herszterg et al., 2014). It is curious that cells furrow symmetrically before polarization, because asymmetric furrowing could apically position the midbody. Perhaps there is no cue to drive asymmetric furrowing to position the midbody at the apical midline before polarization. If polarization requires trafficking of apical components to the midbody, then the apical surface must form first and then be repositioned within the tissue to produce the final organization.

The apical migration of the midbody may represent a new phenomenon during cytokinesis. Midbody migration might occur in other cells undergoing a mesenchyme-to-epithelial transition. Abscission is delayed during midbody migration indicating that daughter cells polarize while connected at the midbody, which might facilitate their reorganization. Indeed, SPD-1 inactivation causes disorganized polarization with AIR-2 reaching the apical surface through a compensatory mechanism. Midbody movements are poorly understood and occur under normal or mutant conditions (Bernabé-Rubio et al., 2016; Herszterg et al., 2013; Morais-de-Sá and Sunkel, 2013; Singh and Pohl, 2014). Septate junction formation drives basal migration of the intercellular canal in *Drosophila* epithelia (Daniel et al., 2018; Wang et al., 2018). Midbody movements could also be regulated by global cortical actin dynamics, which are regulated during cytokinesis (Jordan and Canman, 2012). In the future, it will be important to investigate how the midbody moves to the apical midline.

Cytokinesis in the intestinal lineage undergoes distinct regulatory phases during development. Post-embryonic intestinal divisions involve nuclear but not cytoplasmic divisions leading to the formation of binucleate cells that endoreduplicate to become polyploid (Hedgecock and White, 1985). Each division pattern might require gene expression programs that alter cytokinesis and produce unique proteins delivered to the midbody and apical surface. Transmembrane proteins localized to the tip of the dendrites in amphid sensilla could be delivered during cytokinesis (Heiman and Shaham, 2009). The initial secretory apparatus built during cytokinesis could contribute to the ability of the dendrite cilia to release exosomes (Wang et al., 2014a). Further investigation is required to define the contributions of the midbody to the apical surface in different tissues.

Aurora B regulates several aspects of the specialized cytokinesis that occurs during morphogenesis. Future work will be required to define how Aurora B moves from the midzone microtubules to the apical surface while the MBR is internalized. Aurora B regulates the cytoskeleton during cytokinesis to control cell shape and may have a similar role in lumen formation (Ferreira et al., 2013; Floyd et al., 2013; Goto et al., 2003; Kettenbach et al., 2011). Aurora B regulates abscission timing (Mathieu et al., 2013; Steigemann et al., 2009) and may delay abscission until after midbody migration. Aurora B delays abscission in mouse embryos to allow delivery of adhesion proteins to the midbody (Zenker et al., 2017). Aurora B regulates the central spindle (Bastos et al., 2013), and may contribute to the elongated midzone in E8-E16 gut cells. Aurora B is regulated by a cadherin in zebrafish embryos to organize spindle midzone microtubules (Chen et al., 2018), indicating reciprocal regulation between adhesion and cytokinesis. In the *Xenopus* embryo, altered spindle midzones correlate with changes to furrow ingression and midbody behavior (Kieserman et al., 2008). Although ZEN-4 is rapidly internalized with the midbody in the three tissues, it was previously implicated in morphogenesis of the epidermis and arcade cells independently of cytokinesis (Hardin et al., 2008; Portereiko et al., 2004; Von Stetina et al., 2017). Therefore, further study will be required to understand the role of Aurora B and the spindle midzone during specialized cytokinesis in morphogenesis.

In the sensilla, the centriole is required to form sensory cilia (Dammermann et al., 2009; Nechipurenko et al., 2017; Perkins et al., 1986). Central spindle proteins localize to cilia in *Xenopus* epithelial cells and regulate cilia morphology in the sensilla dendrites (Kieserman et al., 2008; Smith et al., 2011). Aurora B kinase regulates neuronal axon morphology and axonal outgrowth in zebrafish (Gwee et al., 2018). These results indicate a post-mitotic function for various cytokinetic regulators in cilia and cellular architecture. In the intestine, γ-tubulin but not centrioles remain at the apical surface, which forms microvilli rather than cilia (Feldman and Priess, 2012; Leung et al., 1999). Therefore, the midbody and centrosome may contribute different components to regulate the cytoskeletal architecture of the apical surface, which will be a major focus of future studies.

## MATERIALS AND METHODS

### *C. elegans* strains

*C. elegans* strains were maintained with standard protocols. *C. elegans* strains expressing midbody proteins driven by the *pie-1* promoter are listed in Table S3. All temperature-sensitive mutants were obtained from the *Caenorhabditis* Genetics Center.

### CRISPR/Cas9 generation of GFP::AIR-2 and mScarlet::AIR-2 transgenic strains

We followed the CRISPR/Cas9 protocol generated by the Seydoux lab for C-terminus GFP and mScarlet tagging of the *C. elegans air-2* gene (Paix et al., 2015). The repair templates were amplified from pDD282 and pMS050, gifts from Bob Goldstein (Addgene plasmids #66823 and #91826, respectively). The primer sequences were as follows: AIR-2-mScarlet forward: GCAGCAAAAGATTGAAAAAGAAGCAAGTCTTCGAAATCACATGGTCTCCAAGGGAGAGG; AIR-2-mScarlet reverse: AGATGATTGAAAGAAGGACGGGAAAATCAGTAGTTGATCACTTGTAGAGCTCGTCCATTCC; AIR-2-GFP forward: GCAGCAAAAGATTGAAAAAGAAGCAAGTCTTCGAAATCACGGAGCATCGGGAGCC; AIR-2-GFP reverse: AGATGATTGAAAGAAGGACGGGAAAATCAGTAGTTGATCACTTGTAGAGCTCGTCCATTC. The guide RNA sequence is: UUGAAAAAGAAGCAAGUCUU.

### Embryo preparation and imaging

For live imaging, young gravid hermaphrodites were dissected in M9 buffer containing polystyrene microspheres and sealed between two coverslips with petroleum jelly (Pohl and Bao, 2010). Live-cell imaging was performed on a spinning disk confocal system consisting of a Nikon Eclipse inverted microscope with a 60×1.40 NA objective, a CSU-22 spinning disk system, and a Photometrics EM-CCD camera from Visitech International. Images were acquired by Metamorph (Molecular Devices) and analyzed by ImageJ/Fiji Bio-Formats plugins (National Institutes of Health) (Linkert et al., 2010; Schindelin et al., 2012). Whole-embryo live imaging was performed on lattice light-sheet microscopes housed in the Eric Betzig lab, Bi-Chang Chen lab, or the Advanced Imaging Center at HHMI Janelia. The system is configured and operated as previously described (Chen et al., 2014). Briefly, embryos were dissected out and adhered to 5 mm round glass coverslips (Warner Instruments, CS-5R). Samples were illuminated by lattice light sheet using 488 nm or 560 nm diode lasers (MPB Communications) through an excitation objective (Special Optics, 0.65 NA, 3.74-mm WD). Fluorescent emission was collected by detection objective (Nikon, CFI Apo LWD 25XW, 1.1 NA) and detected by a sCMOS camera (Hamamatsu Orca Flash 4.0 v2). Acquired data were de-skewed as previously described (Chen et al., 2014) and deconvolved using an iterative Richardson–Lucy algorithm. Point-spread functions for deconvolution were experimentally measured using 200 nm TetraSpeck beads adhered to 5 mm glass coverslips (Invitrogen, T7280) for each excitation wavelength.

### Immunostaining assay in *C. elegans* embryos

Apical marker staining was performed with the freeze-crack methanol protocol (Leung et al., 1999). Immunostaining with anti-AIR-2 antibodies was performed as described (Schumacher et al., 1998). Primary antibodies and dilutions used were: anti-ERM-1 (1:200; AB_10584795, Developmental Studies Hybridoma Bank); P4A1/PAR-3 (1:200; P4A1, Developmental Studies Hybridoma Bank); DLG-1 (1:200; AB_2617529, Developmental Studies Hybridoma Bank); MH33 (1:150; MH33, Developmental Studies Hybridoma Bank); AIR-2 (1:50; gift from Jill Schumacher, University of Texas MD Anderson Cancer Center, TX, USA). Alexa 488- and 568-conjugated secondary antibodies (1:200-1:1000; A-11059 and A-11004, Invitrogen/Thermo Fisher Scientific) were used in the study. To stain temperature-sensitive mutants, two-cell-stage embryos were dissected from gravid worms, mounted in 10 µl of M9 buffer, and kept cold on ice. The two-cell-stage embryos were incubated at 15°C for 4-7 h until specific stages, then shifted to the restrictive temperature (25°C) for 2-4 h and stained as described above.

### DiI staining in *C. elegans*

DiI staining of wild type and temperature-sensitive mutants was performed as previously described (Tong and Burglin, 2010). Two-cell-stage embryos were incubated at 15°C for 6.5-7 h until they reached the polarized E16 stage, then shifted to the restrictive temperature (25°C) with 1:200 dilution of stock DiI dye solution containing 2 mg/ml DiI in dimethyl formamide for 18-24 h. Hatched larvae were transferred to M9 and washed twice in M9 before mounting in 25 mM levamisole on 2% agar pads for imaging.

### Temperature-shift experiments

Temperature-sensitive mutants were maintained at 15°C. To perform temperature shifts on staged embryos, gravid adults were transferred to a dissection chamber (<4°C), which was precooled in an ice bucket with 20 μl of ice-cold M9 Buffer. Two-cell-stage embryos were quickly transferred (within a 5-10 min time window) via mouth pipette (Aspirator tube assemblies, Sigma-Aldrich) to Fisherbrand Hanging Drop Slides (12-560B, Fisher Scientific) on ice. The slide was placed into a humidified chamber and incubated at 15°C until the appropriate stages were reached, then shifted to 26°C. Incubation times were determined based on *C. elegans* embryonic lineage timing and adjusted according to DAPI staining to ensure each mutant was shifted at a similar stage of embryo development. To inactivate *air-2(or207)*, mutant embryos were incubated for 5 h at 15°C and shifted to 26°C for 3 h to reach the bean stage or for 5 h at 26°C to reach the comma stage. This was the minimum amount of time required to shift embryos to the non-permissive temperature and observe significant cytokinesis defects by the E8-E16 division, indicating significant reduction of AIR-2 function. Most embryos reached the E4-E8 division at the time of the shift. By live imaging we found that there was little disruption of the E4-E8 division under these conditions as *air-2(or207)* embryos (*n*=4/5) have eight normal E8 cells. N2, *spd-1(oj5)* and *zen-4(or153)* embryos were incubated for 4.5 h at 15°C to reach the E4-E8 stage, followed by 3 h at 26°C to reach the bean stage and 5 h at 26°C to reach the comma stage. To shift embryos at the comma stage, *air-2(or207)* embryos were incubated for 12 h at 15°C and N2, *spd-1(oj5)* and *zen-4(or153)* embryos were incubated for 11-11.5 h at 15°C.

## Supplementary Material

Supplementary information
