# Supplementary material for: Aurora B functions at the apical surface after specialized cytokinesis during morphogenesis in C. elegans
Source: Development. 2020 Jan 8;147(1):dev181099. doi: 10.1242/dev.181099 (PMC6983721; doi:10.1242/dev.181099)
Supplement: Supplementary information [file develop-147-181099-s1.pdf]

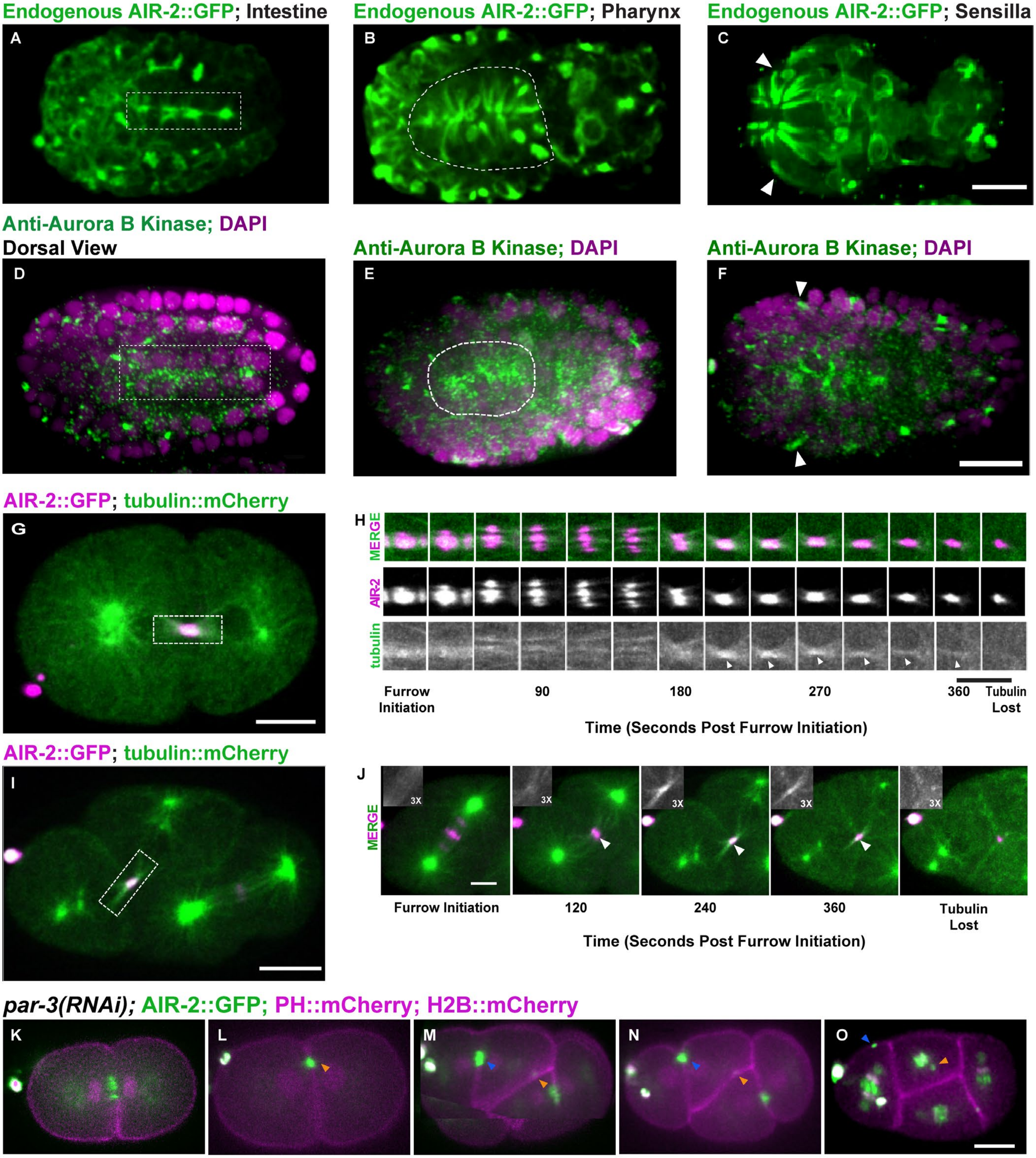

Figure S1. Aurora B kinase during embryo development.

Endogenously tagged AIR-2::GFP shows apical localization in E16 intestinal cells (dashed box in A), polarized pharyngeal cells (dashed shape in B), and elongating sensilla neurons (arrowheads, C). (D) AIR-

Development • Supplementary information

2 staining also shows apical surface of polarized E16 intestine (D, rectangle), pharynx (E, dotted circle) and at the apical cluster of the amphid sensilla (F, arrowheads). (G-H) AIR-2::GFP (magenta) and tubulin::mCherry (green) colocalize at the central spindle during cytokinesis in the first cell division. AIR-2::GFP persists at the midbody remnant after microtubules are lost, which indicates rapid abscission timing (H). (I-J) AIR-2::GFP (magenta) and tubulin::mCherry (green, inset) colocalize on the AB central spindle and midbody (arrowhead). Microtubules quickly disappear indicating abscission (J). (K-O) In *par-3(RNAi)* embryos, the P0 furrow becomes more asymmetric causing offset midbody positioning (L, orange arrowhead). The AB furrow is less asymmetric and ingresses in the opposite direction (M, blue arrowhead indicates misplaced AB midbody). (O) Midbody remnants are inherited randomly. Scale bar, 10 µm.

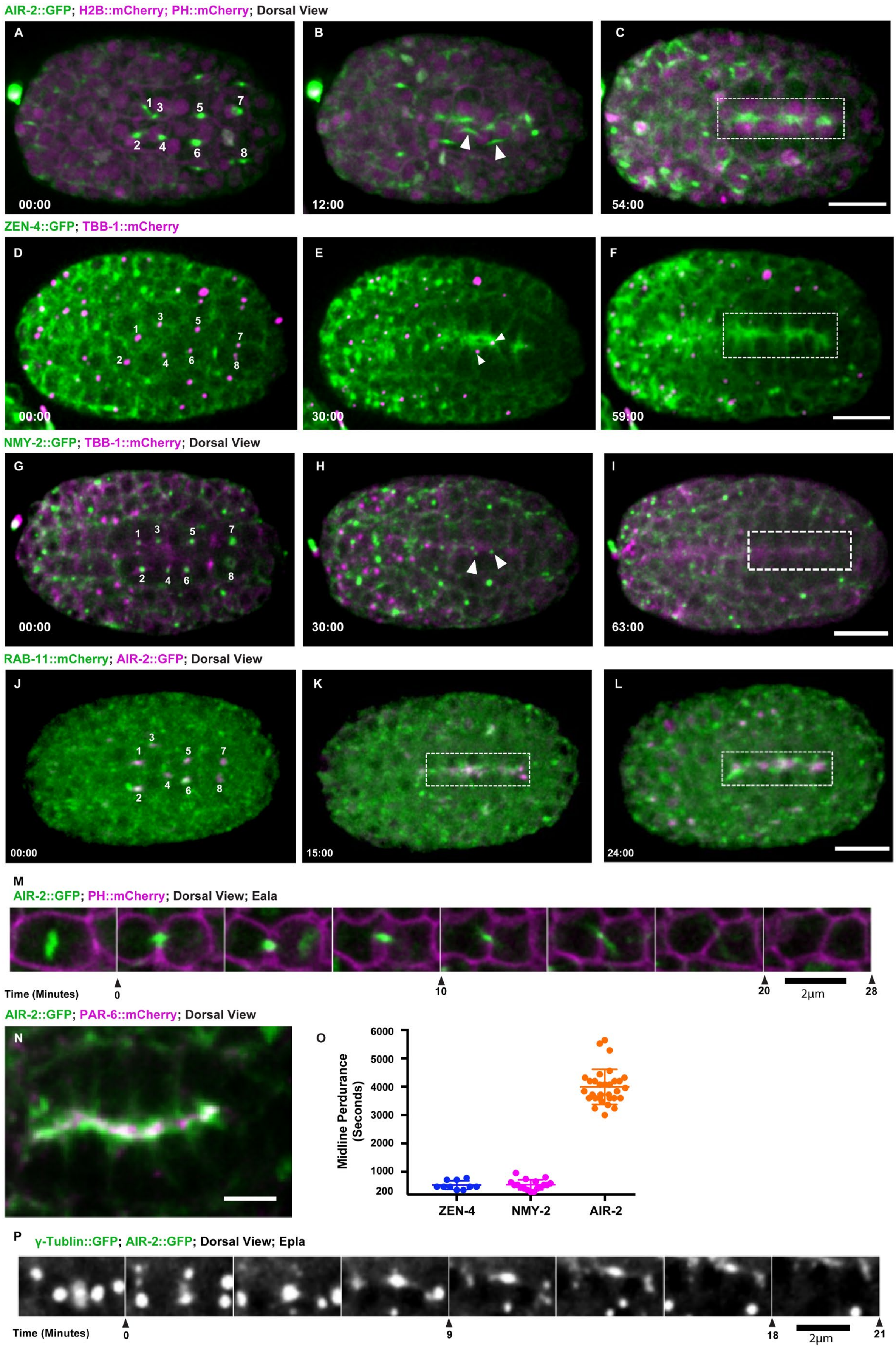

Development • Supplementary information

Figure S2. Cytokinesis in the intestine epithelia

Cytokinesis in the E8-E16 division. (A-C) AIR-2::GFP (green, H2B::mCherry and PH::mCherry are magenta) localizes to midbodies (A, numbered 1-8) that migrate toward the apical midline (B). AIR-2:GFP remains at the apical surface after polarization (C). (D-F) ZEN-4::GFP (magenta, TBB-1::mCherry in green) appears on midbodies (D, numbered 1-8) that migrate to the midline (E) and are quickly removed (F, rectangle box). (G-I) NMY-2 (green, microtubules in magenta) localizes to midbody rings (labeled 1-8 in G) that move to the midline (arrowheads, H) but do not persist (rectangle box in I). (J-L) RAB-11::mCherry (green) and AIR-2::GFP (magenta) colocalize on midbodies (labeled as 1-8 in J) as they migrate to the midline (K) and persist well after polarization is complete (L, rectangle). (M) Midbody from Eala at the E8-E16 division migrates towards the midline but the AIR-2 signal diminishes (green, PH::mCherry in magenta). (N) PAR-6::mCherry (magenta) colocalizes with AIR-2::GFP (green) at the apical midline. (O) Quantification of midline perdurance of different midbody components (measured from the end of furrowing to internalization or loss of signal) shows that AIR-2 remains at the apical surface after the midbody remnant is internalized. Error bars represent the standard deviation. (P) Image series showing the simultaneous movement of AIR-2::GFP (red arrowhead) on the midbody and γ-tubulin::GFP (white arrowheads) on centrosomes to the apical surface. Time shown in minutes: seconds. Scale bar, 10 μm.

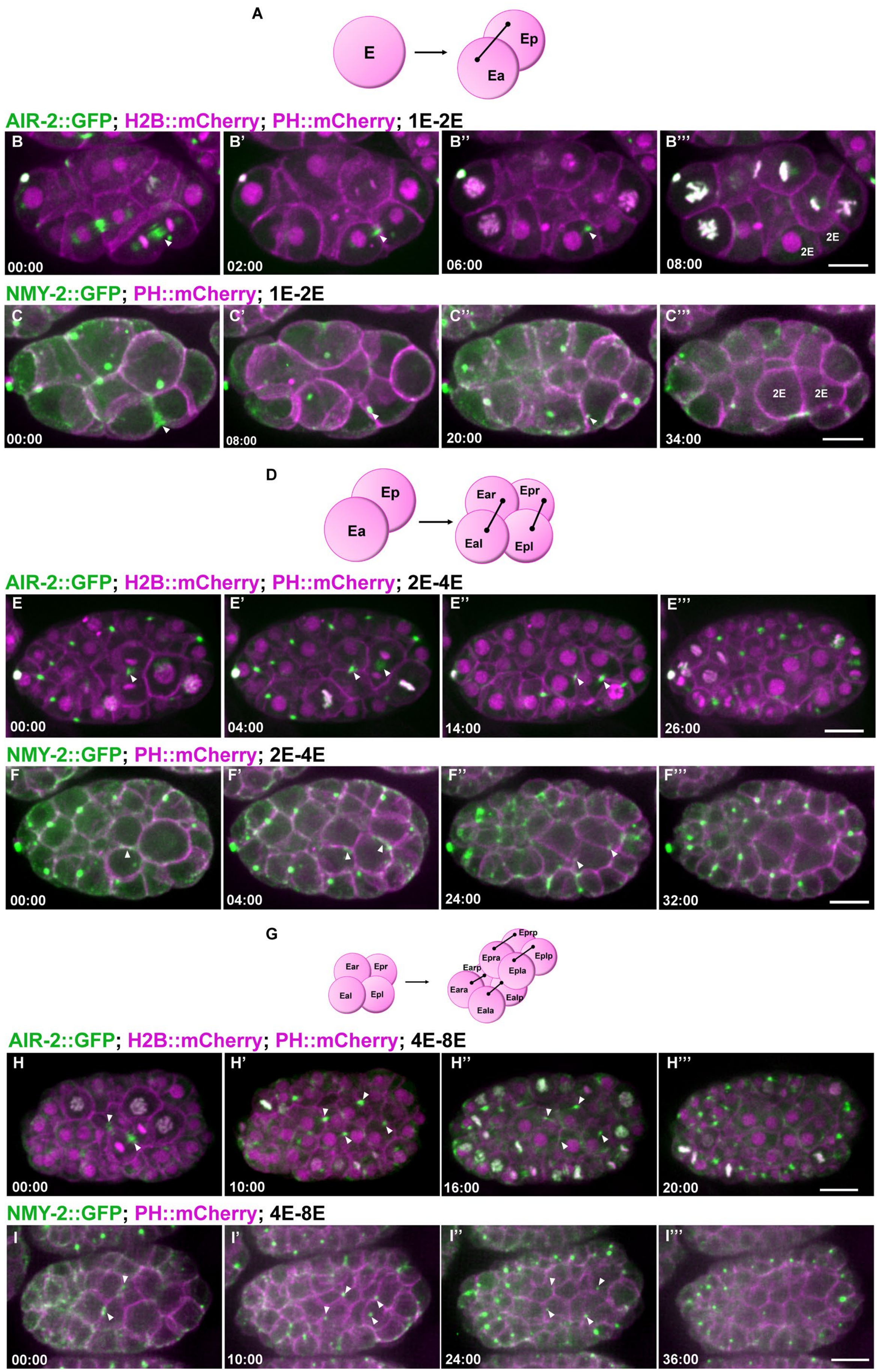

Development • Supplementary information

Figure S3. Midbody dynamics during divisions of the early E lineage

(A) Diagram of the E-E2 division. Dynamics of Aurora B (B) and NMY-2 (C) show formation of the midbody (arrowheads) and rapid internalization. A similar pattern is also observed during the E2-E4 divisions (D-F) and the E4-E8 divisions (G-I), demonstrating normal midbody dynamics in the first three E lineage embryonic divisions. Scale bar, 10 µm.

Development • Supplementary information

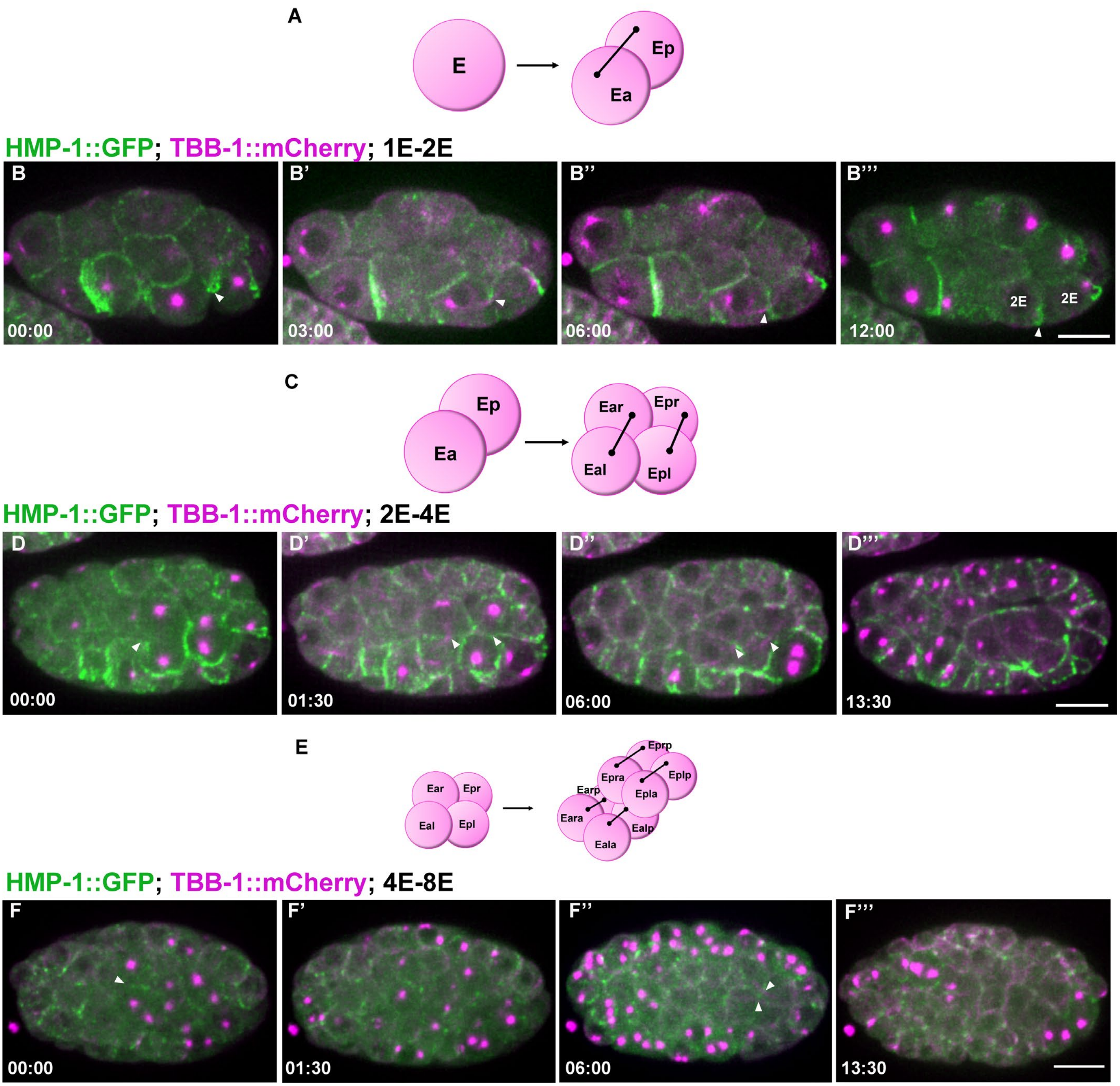

Figure S4. Localization of α-catenin during the E lineage cell divisions

(A) Diagram of the E-E2 division. HMP-1::GFP localizes to the furrow and membrane adjacent to the midbody during cytokinesis (arrowheads) and remains there after spindle midzone microtubules disappear. (C) Diagram of the E2-E4 division. HMP-1::GFP can be observed in the cortex of the dividing cells (arrowhead, D) and localizes along the furrow membrane and adjacent to the midbody (arrowheads, D-D”). (E) Diagram of the E4-E8 divisions. (F) Arrowheads indicate accumulation of HMP-1::GFP near the midbody during cytokinesis. HMP-1::GFP does not accumulate at the midline until the end of the E8-E16 division. Scale bar, 10 µm.

Development • Supplementary information

A Cytokinesis Completion — One Cytokinesis Failure — Two Cytokinesis Failure

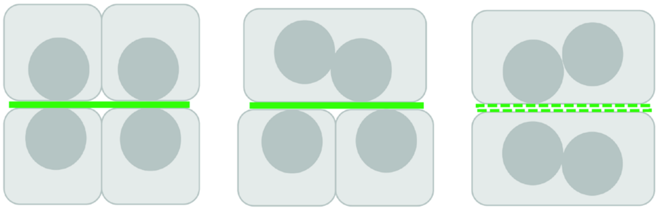

Defects in Microtubule Accumulation

B

Genotype	Total Embryo	Total 8E Cells	Cytokinesis Failure	Nuclei Polarization Defects	Chromosome Segregation Defects	Microtubule Accumulation Defects
WT (N2)	8	34	0/34 (0%)	0/34 (0%)	0/34 (0%)	0/8 (0%)
air-2(or207ts)	11	66	18/66 (27.3%)	17/66 (25.8%)	3/66 (4.5%)	5/11 (45.5%)

C

Genotype	Pair of 8E Cells	Pair Number	One Cytokinesis Failure	Microtubule Discontinuous	Two Cytokinesis Failure	Microtubule Discontinuous
WT (N2)	Ea(r/l)a	8	0/8 (0%)	0/0 (0%)	0/8 (0%)	0/0 (0%)
	Ea(r/l)p	8	0/8 (0%)	0/0 (0%)	0/8 (0%)	0/0 (0%)
	Ep(r/l)a	8	0/8 (0%)	0/0 (0%)	0/8 (0%)	0/0 (0%)
air-2(or207ts)	Ea(r/l)a	11	5/11 (45.5%)	1/5 (20%)	0/11 (0%)	0/0 (0%)
	Ea(r/l)p	11	6/11 (54.4%)	0/6 (0%)	0/11 (0%)	0/0 (0%)
	Ep(r/l)a	11	3/11 (27.3%)	1/3 (33.3%)	2/11 (18.2%)	1/2 (50.0%)

Defects in Adhesion Complex Accumulation

D

Genotype	Total Embryo	Total 8E Cells	Cytokinesis Failure	Nuclei Polarization Defects	Chromosome Segregation Defects	HMP-1 Accumulation Defects
WT (N2)	5	30	0/30 (0%)	0/30 (0%)	0/30 (0%)	0/5 (0%)
air-2(or207ts)	12	72	30/72 (41.7%)	25/72 (34.7%)	0/72 (0%)	6/12 (50.0%)

E

Genotype	Pair of 8E Cells	Pair Number	One Cytokinesis Failure	HMP-1 Discontinuous	Two Cytokinesis Failure	HMP-1 Discontinuous
WT (N2)	Ea(r/l)a	5	0/5 (0%)	0/0 (0%)	0/5 (0%)	0/0 (0%)
	Ea(r/l)p	5	0/5 (0%)	0/0 (0%)	0/5 (0%)	0/0 (0%)
	Ep(r/l)a	5	0/5 (0%)	0/0 (0%)	0/5 (0%)	0/0 (0%)
air-2(or207ts)	Ea(r/l)a	12	3/12 (45.5%)	0/3 (0%)	3/12 (0%)	3/3 (100%)
	Ea(r/l)p	12	6/12 (54.4%)	0/6 (0%)	1/12 (8.3%)	0/1 (0%)
	Ep(r/l)a	12	4/12 (33.3%)	0/4 (0%)	4/12 (33.3%)	3/4 (75.0%)

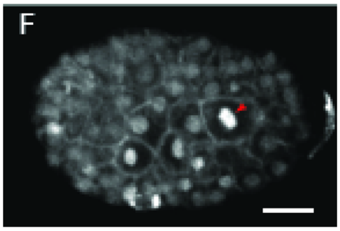

H2B::mCherry; PH::mCherry; air-2(or207ts)

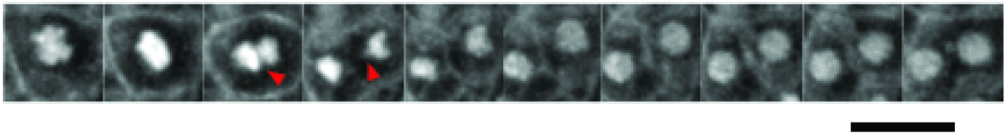

Figure S5. Quantification of defects observed in *air-2(or207ts)* mutant embryos

(A) Diagram of failure patterns of pairs of E8-E16 divisions on opposite sides of midline and observed consequence on apical microtubule accumulation (green). (B) Quantification of defects in *air-2(or207ts)* mutant E8-E16 divisions showing significant cytokinesis failures where most cells that failed also have nuclear polarization defects. Of the 11 embryos observed, five have apical microtubule accumulation defects. The low incidence of chromosome segregation defects are consistent with weak Aurora B inactivation. (C) Quantification of individual cell division failures and incidence of discontinuous microtubule accumulation at the apical midline after polarization. (D) Quantification of defects in *air-2(or207ts)* mutant E8-E16 divisions showing significant defects in HMP-1::GFP accumulation with cytokinesis failures. (E) Quantification of individual cell division failures and incidence of discontinuous adhesion accumulation at the apical midline after polarization. (F) An example of an intestinal cell in an Aurora B mutant embryo showing lagging chromosome segregation (red arrowheads).

Development • Supplementary information

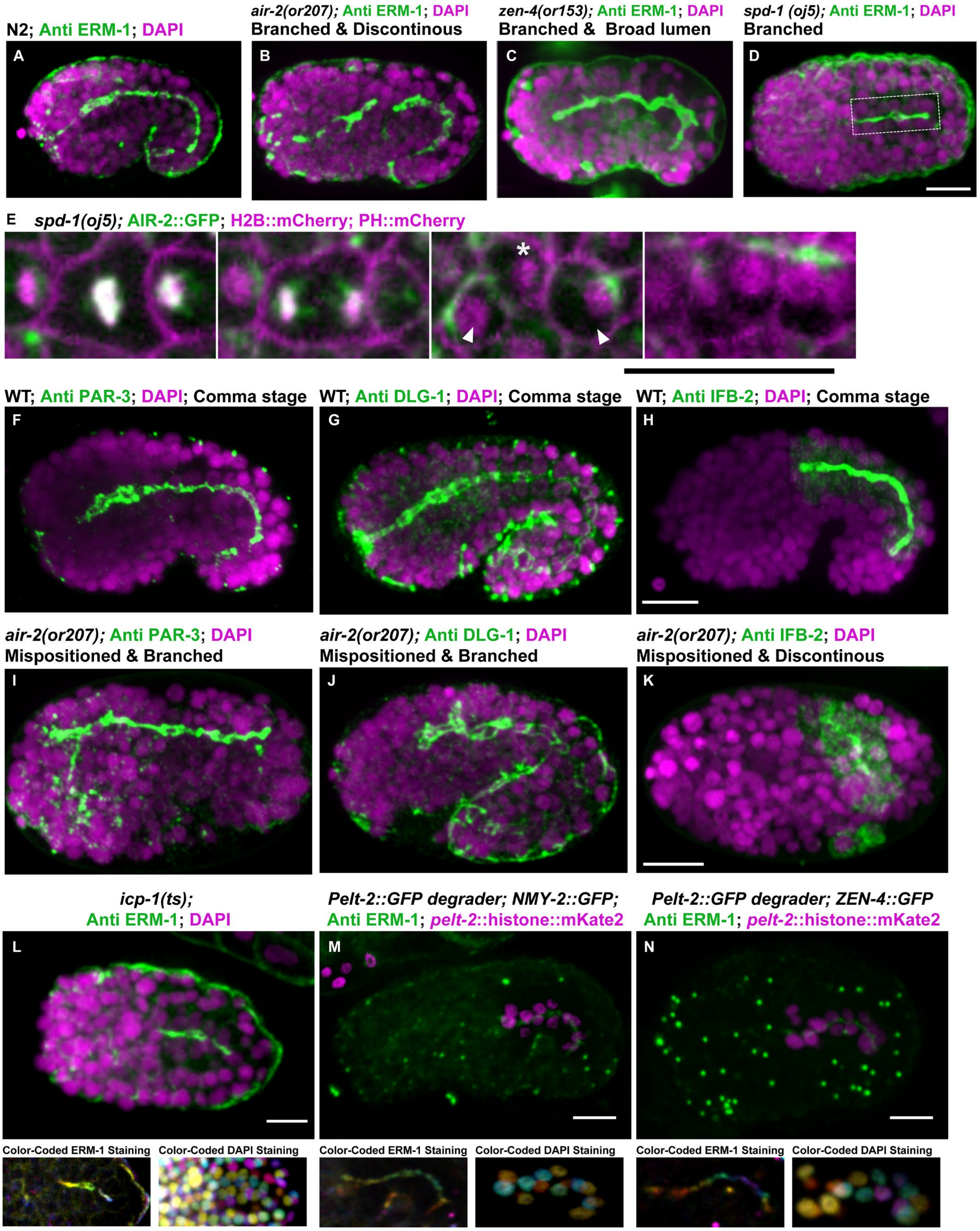
N2; Anti ERM-1; DAPI
air-2(or207); Anti ERM-1; DAPI
Branched & Discontinous
zen-4(or153); Anti ERM-1; DAPI
Branched & Broad lumen
spd-1 (oj5); Anti ERM-1; DAPI
Branched
A
B
C
D
E
spd-1(oj5); AIR-2::GFP; H2B::mCherry; PH::mCherry
*
WT; Anti PAR-3; DAPI; Comma stage
WT; Anti DLG-1; DAPI; Comma stage
WT; Anti IFB-2; DAPI; Comma stage
F
G
H
air-2(or207); Anti PAR-3; DAPI
Mispositioned & Branched
air-2(or207); Anti DLG-1; DAPI
Mispositioned & Branched
air-2(or207); Anti IFB-2; DAPI
Mispositioned & Discontinous
I
J
K
icp-1(ts);
Anti ERM-1; DAPI
Pelt-2::GFP degrader; NMY-2::GFP;
Anti ERM-1; pelt-2::histone::mKate2
Pelt-2::GFP degrader; ZEN-4::GFP
Anti ERM-1; pelt-2::histone::mKate2
L
M
N
Color-Coded ERM-1 Staining
Color-Coded DAPI Staining
Color-Coded ERM-1 Staining
Color-Coded DAPI Staining
Color-Coded ERM-1 Staining
Color-Coded DAPI Staining

Figure S6. Cytokinesis mutants have disrupted intestinal and pharyngeal tubulogenesis

(A) ERM-1 stains the apical lumen of the gut and pharynx in wild-type (N2) embryos at the comma stage. Gut lumen defects are observed in (B) *air-2(or207),* (C) *zen-4(or153)* and (D) *spd-1(oj5)* embryos. (E) In *spd-1(oj5)* embryos, AIR-2::GFP does not accumulate at the spindle midzone and instead appears on spindle poles (arrowheads). A neighboring cell comes between E16 sisters (asterisk), but AIR-2 eventually moves to the apical midline with the poles. (F) PAR-3, (G) DLG-1 and (H) IFB-2 localize to the lumen in N2 embryos. (I-K) In *air-2(or207)* mutants, apical surface markers localize to distorted and mispositioned lumens. (L) Lumen defects are observed in *icp-1(or663ts)* mutant embryos. (M-N) Tissue-specific degradation of endogenously tagged NMY-2::GFP (M) or ZEN-4::GFP (N) in the gut does not cause significant widening of the apical lumen. Scale bar, 10 μm.

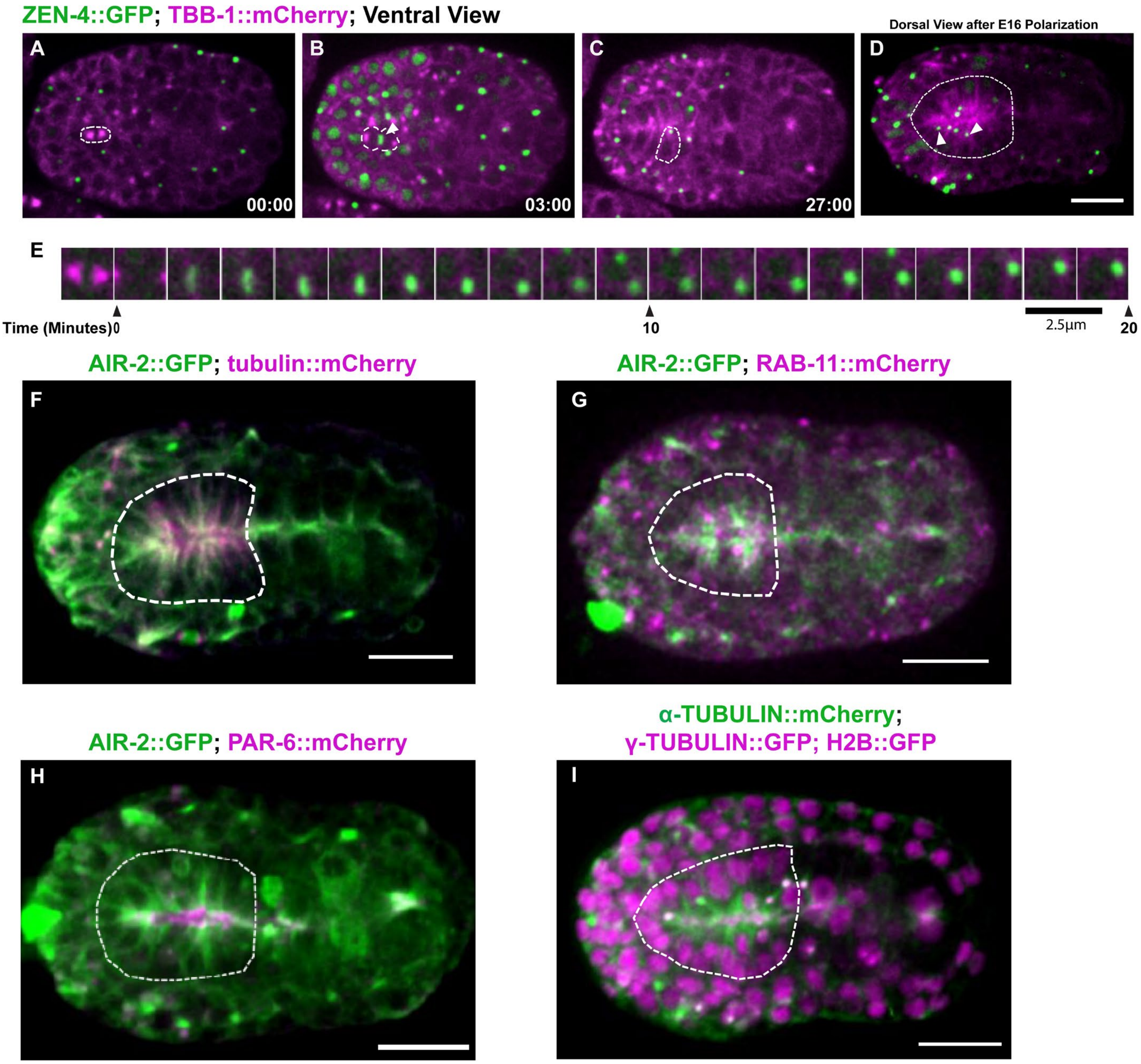

Figure S7. Cytokinesis in Pharynx Precursor Cells

(A-E) Centralspindlin ZEN-4::GFP dynamics in PPC divisions. Midbody remnants labeled with ZEN-4::GFP migrate to the midline (arrowheads, C) and are rapidly internalized and eventually degraded (arrowheads in D show internalized ZEN-4 labeled midbodies). AIR-2::GFP (F, G green) colocalized with Tubulin::mCherry (F, magenta) and partially colocalized with RAB-11::mCherry (G, magenta) at the apical surface of the polarized pharynx (dotted circle). AIR-2::GFP (H, green) partially co-localized with PAR-6::mCherry (H, magenta) at the apical surface of the pharynx (dotted circle). (I) The apical surface of the pharynx accumulates γ-tubulin::GFP (magenta in merge, microtubules in green). Scale bar, 10 µm.

Development • Supplementary information

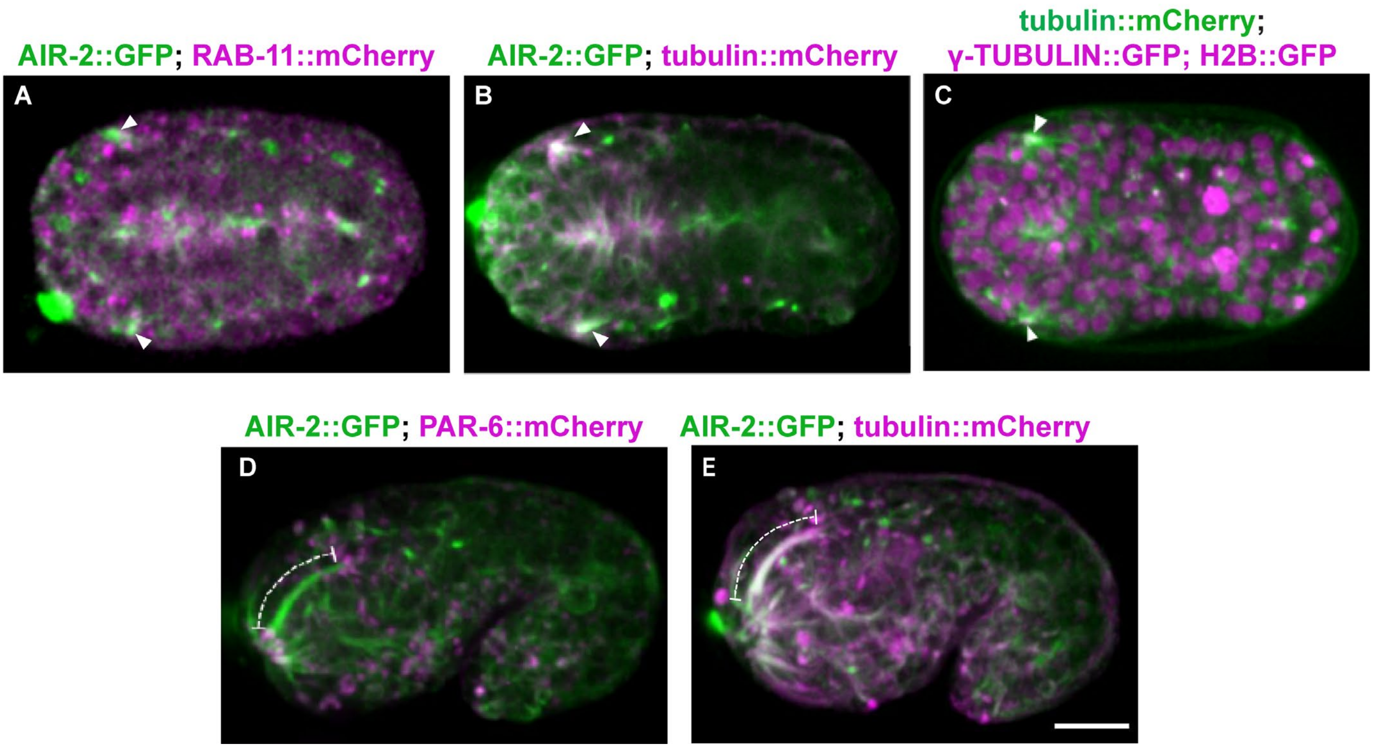

Figure S8. Midbody components label dendrites of sensilla neurons

Aurora B kinase (green) colocalizes with (A) RAB-11::mCherry (magenta) and (B) tubulin::mCherry (magenta) at the apical surface of sensilla (white arrowheads). (C) γ-tubulin::GFP (magenta) localizes at the apical cluster with microtubules (green). (D) AIR-2::GFP (green) localizes along the dendrite extension (dashed line) and PAR-6::mCherry is observed at the tip of the extension (arrowhead), indicating it is the apical domain. (E) AIR-2::GFP (green) and microtubules (tubulin::mCherry, magenta) colocalize along the length of the extended dendrite (dashed line). Scale bar, 10 µm.

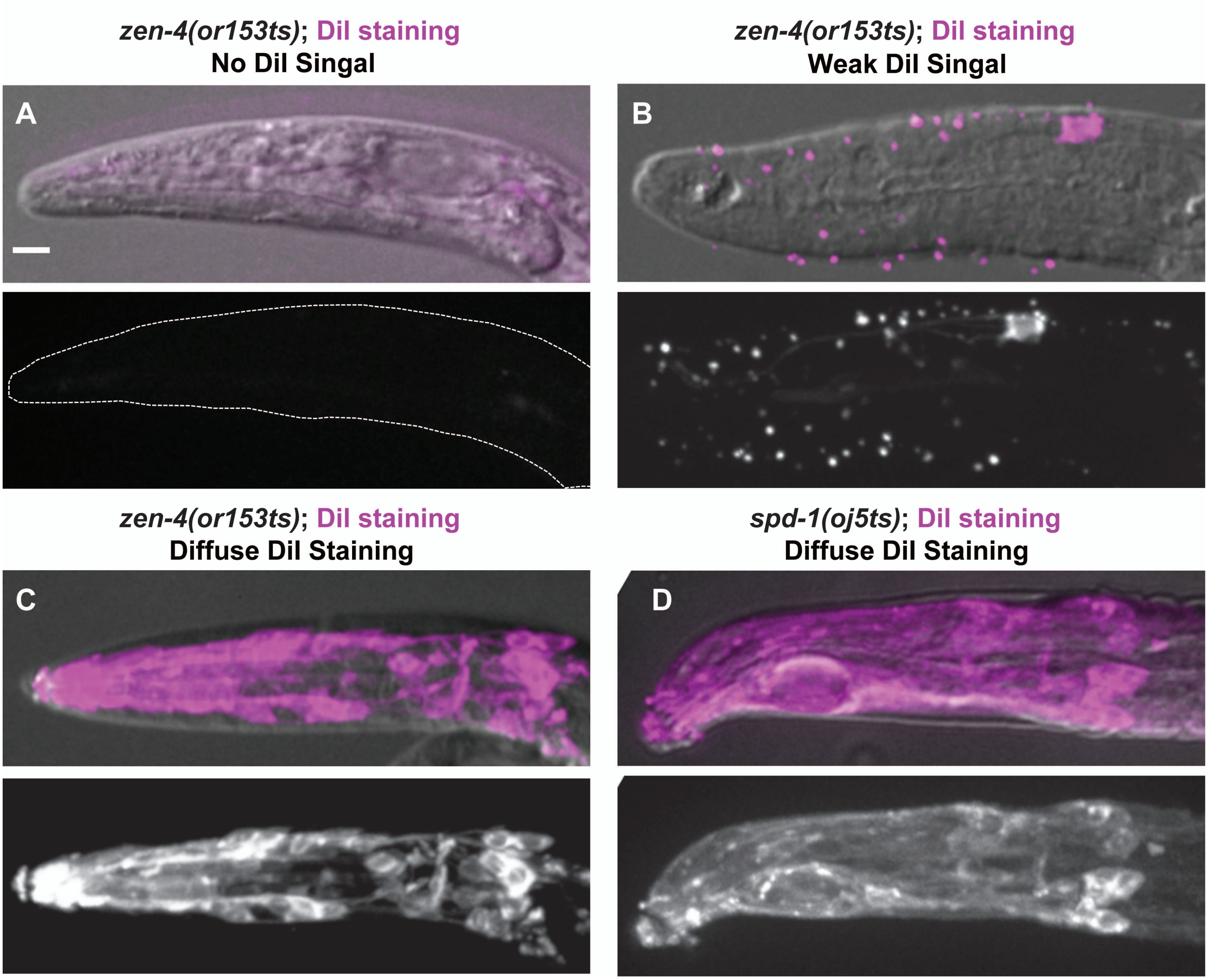

Figure S9. Cytokinesis mutants have disrupted sensilla neuron morphogenesis

Visualizing dendrite and neuron morphology by DiI staining in surviving larvae at non-permissive temperature. (A-C) Hatched *zen-4(or153ts)* mutant larvae display a variety of defects including No-DiI signal (A), weak (B) and Diffuse (C) DiI signal. *spd-1(oj5)* mutant embryo also show sensilla neuron morphogenesis defects including diffuse DiI staining pattern (D).

Development • Supplementary information

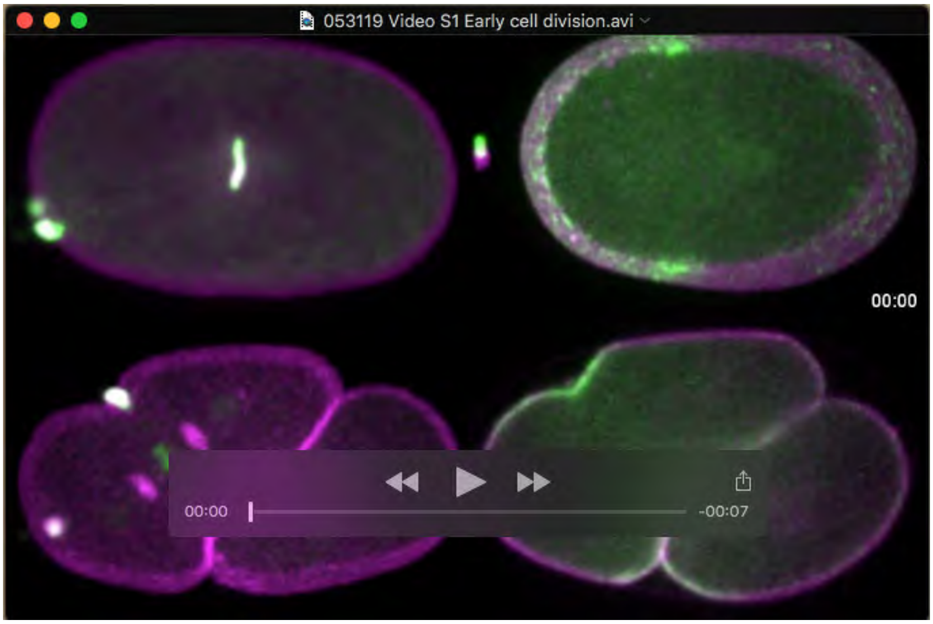

Movie 1. Cytokinesis in the first two mitotic divisions

Cytokinesis in the first (top row) or second (bottom row) division in embryos expressing AIR-2::GFP (green, left, with H2B::mCherry and PH::mCherry in magenta) or NMY-2::GFP (green, right, with PH::mCherry in magenta). White arrowheads indicate first midbody that is internalized by AB and red arrowheads indicate the AB midbody, which is engulfed by EMS. Images are maximum Z projections of 15 central planes spaced 1µm apart taken every 90 seconds. Playback rate is 2 frames/second.

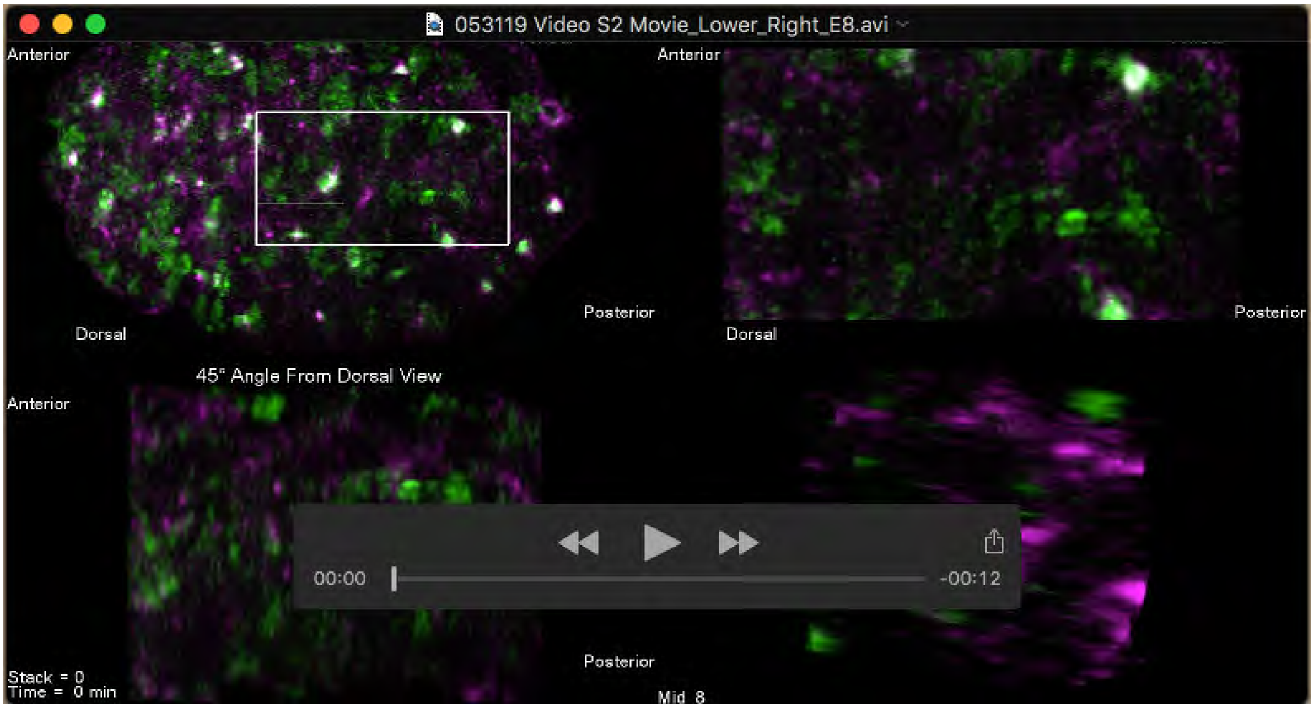

Movie 2. Lattice Light Sheet Imaging of Intestinal Cytokinesis

E8-E16 intestinal cell divisions in embryos expressing AIR-2::mScarlet (green) with NMY-2::GFP (magenta) imaged with lattice light sheet microscopy. A ventral view of the whole embryo (top left) is shown with a region of interests (white box) highlighted in the top right panel. Rotated views shown a dorsal view (bottom right) and a 45 degrees from a dorsal view (bottom left) and show that AIR-2::GFP accumulates to a narrow band around the emerging apical surface. Images are maximum Z projections of images acquired every 60 seconds.

Development • Supplementary information

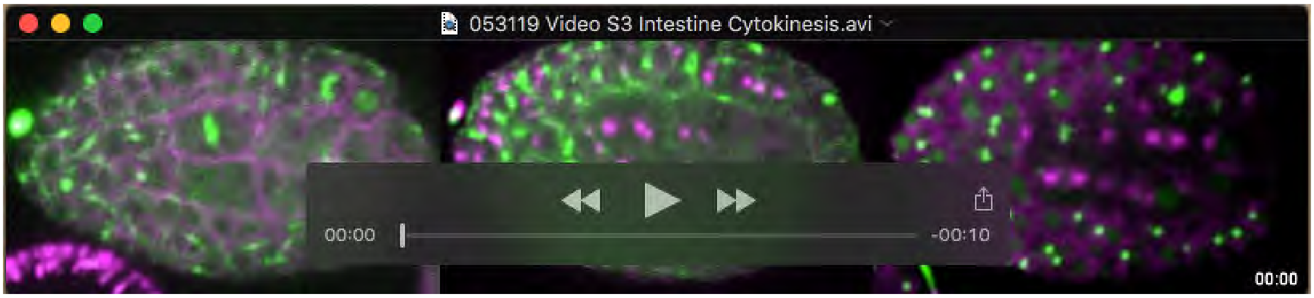

Movie 3. Cytokinesis in the intestine epithelia

E8-E16 intestinal cell division in embryos expressing AIR-2::GFP (green, left, PH::mCherry in magenta), NMY-2::GFP (green, middle, tubulin::mCherry in magenta) or ZEN-4::GFP (green, right, tubulin::mCherry in magenta). The midbodies (indicated by arrowheads) form and move toward the apical surface. ZEN-4::GFP and NMY-2::GFP rapidly disappear, while AIR-2::GFP persists at the apical midline. Images are maximum Z projections of 10 planes spaced 1µm apart, taken every 60 seconds. Playback rate is 6 frames/second.

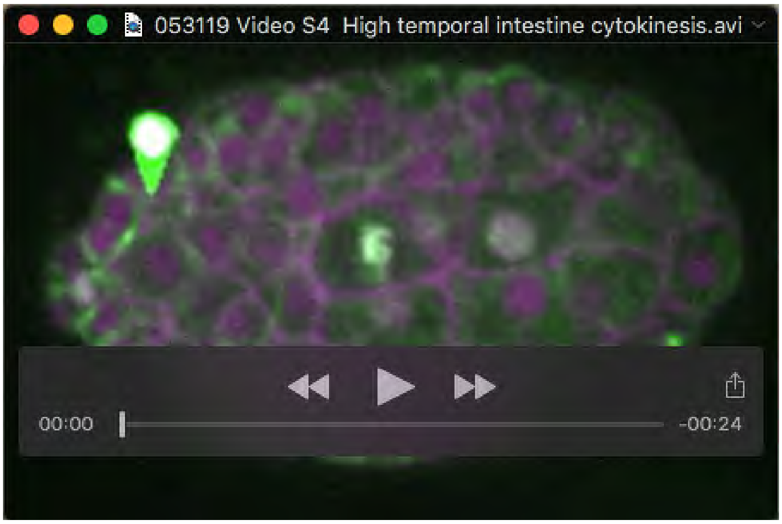

Movie 4. High temporal resolution of midbody dynamics in the intestine

Imaging Earp cell division with high temporal resolution in an embryo expressing AIR-2::GFP (green, PH::mCherry in magenta) shows the lengthening of the central spindle and midbody migration event. Single plane images were acquired every 10 seconds. Playback rate is 15 frames/second.

Development • Supplementary information

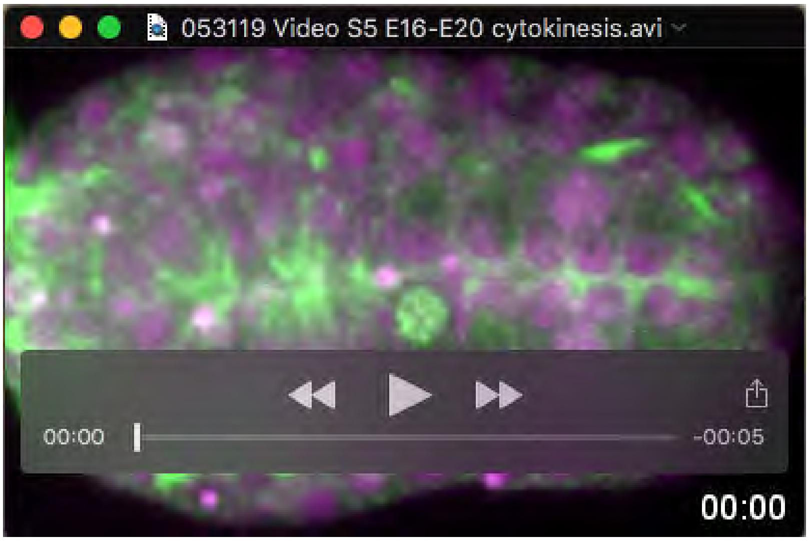

Movie 5. Cytokinesis in E16 to E20 cell division

E16-E20 cell division in embryos expressing AIR-2::GFP (green) with PH::mCherry and H2B::mCherry (magenta). Images are Maximum Z projections of 15 z planes 1 µm apart that were acquired every 90 seconds. Playback rate is 6 frames/second.

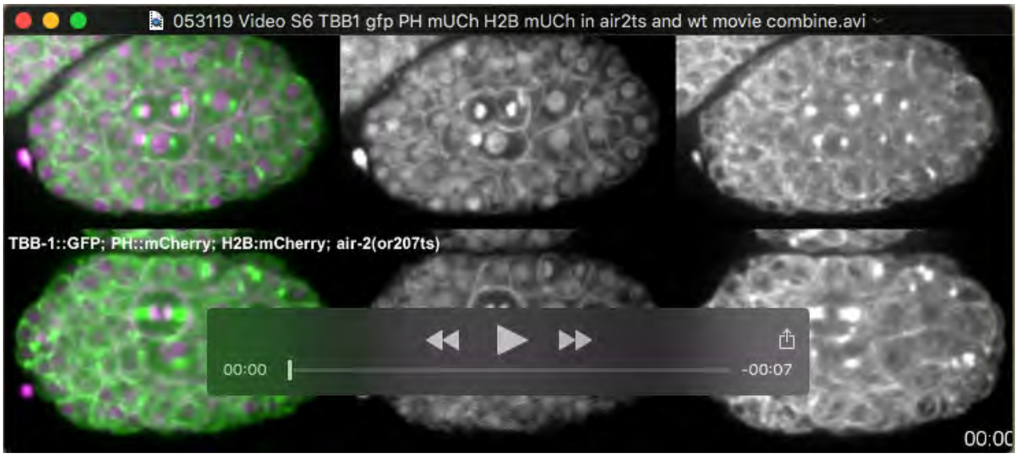

Movie 6. Microtubule dynamics during E8-E16 in Aurora B mutants.

WT (top row) and *air-2(or207)* (bottom row) mutant embryos expressing TBB-1::GFP (green in merge, right movie) and PH::mCherry and H2B::mCherry (middle, magenta in merge). Spindle midzone microtubules in WT embryos move to the midline where microtubules accumulate. In Aurora B mutant embryos, spindle midzone microtubules are diminished and cells that fail cytokinesis (right pair of gut cells) delay microtubule accumulation compared with the cells that do not fail. Images are maximum Z projections of 10-15 z planes 1 µm apart that were acquired every 60 seconds. Playback rate is 6 frames/second.

Development • Supplementary information

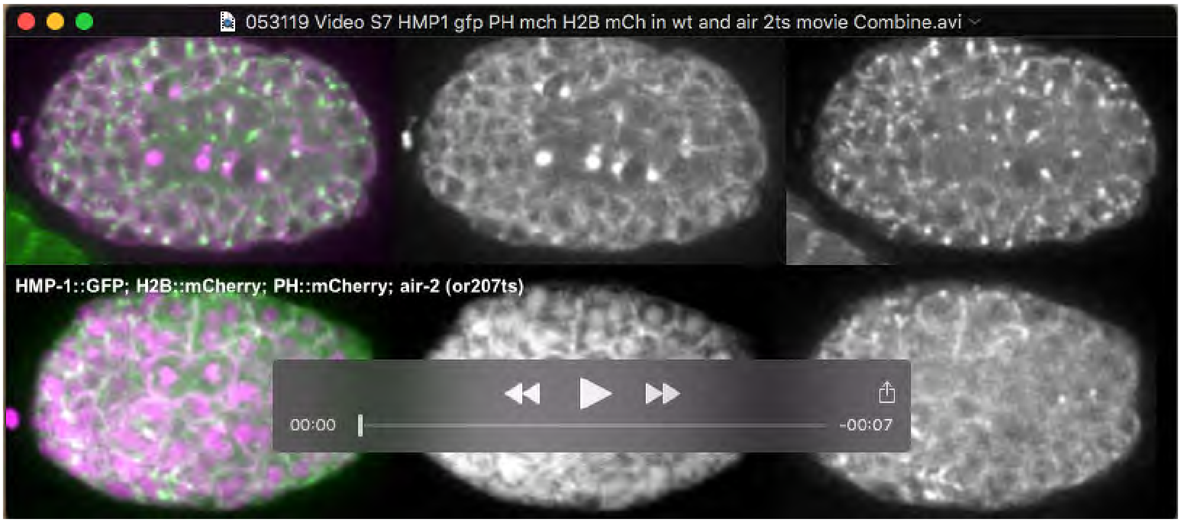

Movie 7. Aurora B regulates adhesion dynamics during E8-E16 epithelial polarization.

WT (top row) and *air-2(or207)* (bottom row) mutant embryos expressing HMP-1::GFP (green in merge, right movie) and TBB-1::mCherry (top middle, magenta in upper left merge) or PH::mCherry and H2B::mCherry (bottom middle, magenta in lower left merge). In WT, HMP-1::GFP accumulates at the furrow and midbody as it migrates to the apical surface where it accumulates during polarization. In Aurora B mutant embryos, HMP-1::GFP is reduced in the furrow and midbody and cells that fail cytokinesis (left pair of gut cells) delay adhesion accumulation at the midline. Images are maximum Z projections of 10-15 z planes 1 µm apart that were acquired every 60 seconds. Playback rate is 6 frames/second.

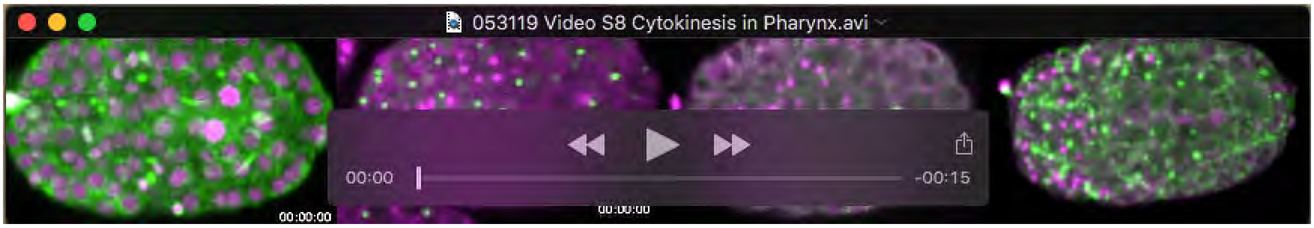

Movie 8. Cytokinesis in the pharynx from ventral views.

Cell division in pharyngeal precursor cells in embryos expressing AIR-2::GFP (green, left, H2B::mCherry in magenta), ZEN-4::GFP (green, middle left, TBB-1::mCherry in magenta), HMP-1::GFP (green, middle right, TBB-1::mCherry in magenta) or NMY-2::GFP (green, right, TBB-1::mCherry in magenta). Midbodies (white arrowheads) migrate toward pharyngeal midline after forming centrally between daughter cell pairs. Images are maximum Z projections of 10-15 z planes 1 µm apart that were acquired every 90 seconds. Playback rate is 6 frames/second.

Development • Supplementary information

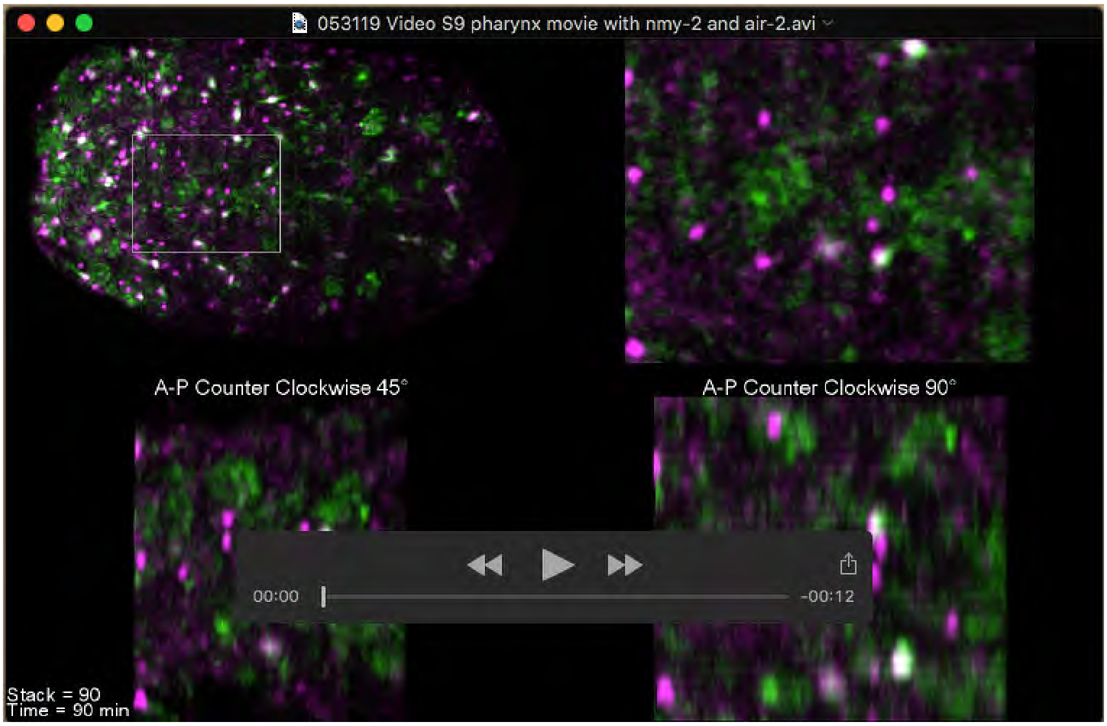

Movie 9. Lattice light sheet imaging of pharyngeal precursor cell divisions

Dorsal view (top left) of lattice light sheet microscopy of pharyngeal precursor cell cytokinesis in embryos expression AIR-2::mScarlet (green) with NMY-2::GFP (magenta) shows migration of midbodies towards the apical midline. The pharyngeal precursor cells in the highlighted region (top right) are shown in zoomed in view of a 45-degree (bottom left) and 90-degree (bottom right) rotation along the anterior-posterior axis of the embryo. Images are max intensity projections of images acquired every 60 seconds.

Development • Supplementary information

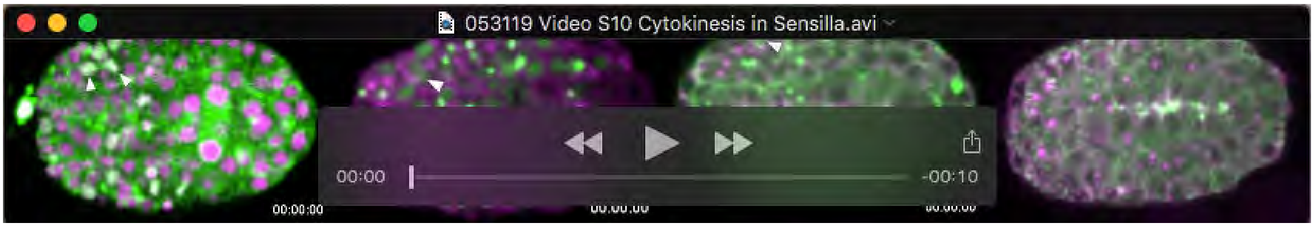

Movie 10. Cytokinesis in the sensilla dendrite development

Cell division in sensilla precursor cell divisions in embryos expressing AIR-2::GFP (green, left, H2B::mCherry in magenta), ZEN-4::GFP (green, middle left, TBB-1::mCherry in magenta), NMY-2::GFP (green, middle right, TBB-1::mCherry in magenta), or HMP-1::GFP (green, right, TBB-1::mCherry in magenta). Midbodies (white arrowheads) migrate into an apical cluster. Images are maximum Z projections of 10 z planes 1 µm apart that were acquired every 90 seconds. Playback rate is 6 frames/second.

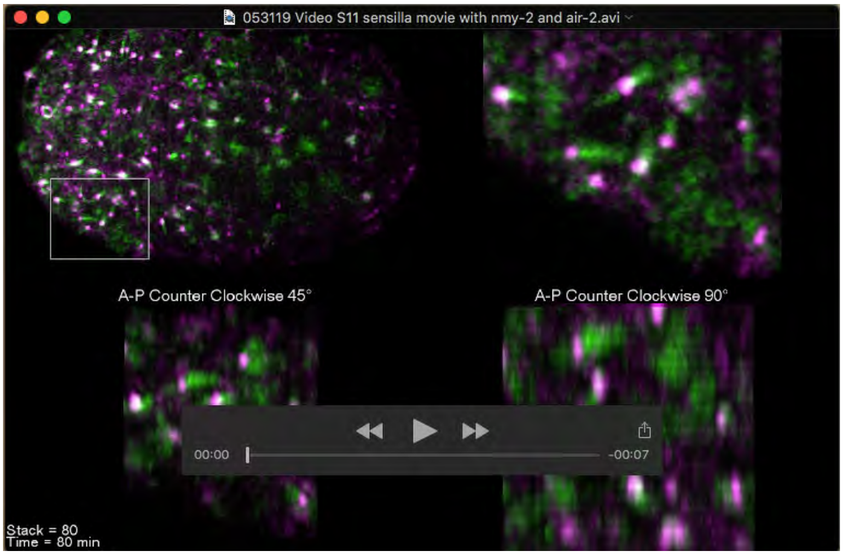

Movie 11. Lattice Light Sheet imaging of Sensilla Precursor Cell Division

Dorsal view of embryos expressing midbody flank marker AIR-2::mScarlet (green) with NMY-2::GFP (magenta) during the sensilla precursor divisions (top left). The sensilla precursor cells are highlighted in the white box (top right) and zoomed in views of this region at a 45-degree (bottom left) and 90-degree (bottom right) rotation along the anterior-posterior axis of the embryo are also shown. Images are maximum intensity Z projections of images acquired every 60 seconds.

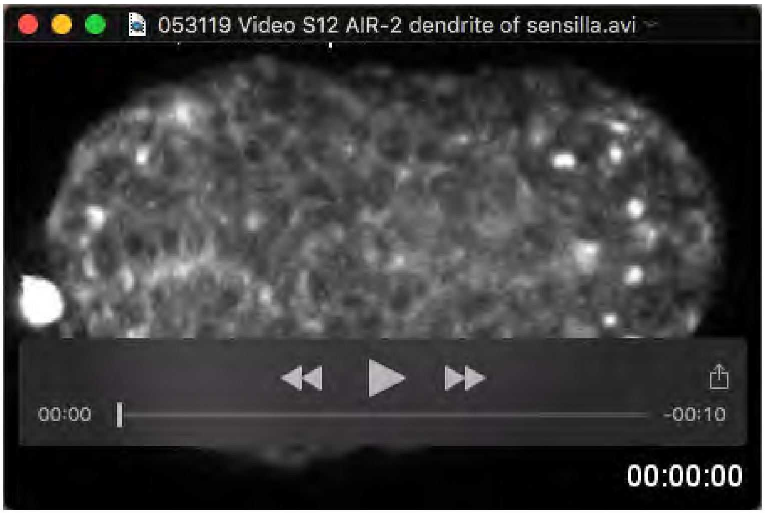

Movie 12. Dendrite Extension of sensilla neurons

Embryo expressing AIR-2::GFP from the apical cluster stage of amphid polarization through the process of dendrite extension. Images are maximum Z projections of 7 planes 1 µm apart viewed from the ventral aspect and acquired every 90 seconds. Playback rate is 6 frames/second.

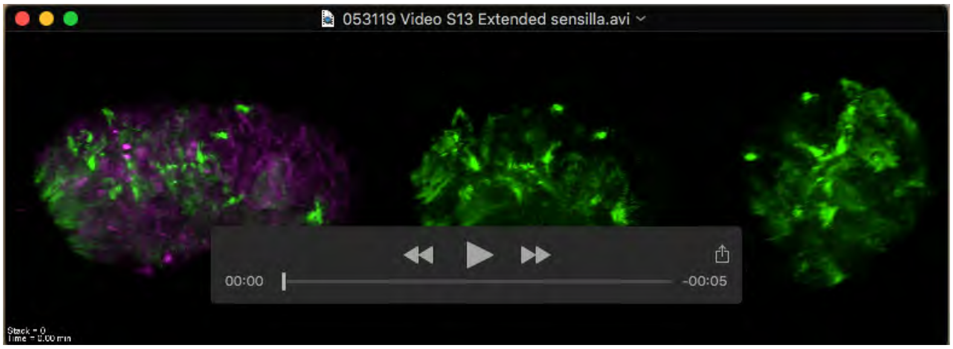

Movie 13. Dendrite Extension of sensilla neurons

Embryo expressing AIR-2::mScarlet (green) with NMY-2::GFP (magenta) during amphid neuron dendrite extension. A 45 degree (middle panel) and a 90 degree (right panel) rotated view of the mouth of the animal are also shown. Images are maximum Z projections of images acquired every 60 seconds.

Table S1. Hatch rate of temperature sensitive mutants dissected at the two cell stage.

Stage Before Shifting	Genotype	Hatch Rate % (Hatch Embryos/Total)
15 °C Forever	N2	100% (32/32)
	air-2(or207)	53.6% (37/69)
	zen-4(or153)	100% (28/28)
	spd-1(oj5)	100% (35/35)
E4-E8	N2	100% (26/26)
	air-2(or207)	6.3% (2/32)
	zen-4(or153)	0% (0/57)
	spd-1(oj5)	100% (48/48)
E8-E16	N2	100% (45/45)
	air-2(or207)	14.4% (13/90)
	zen-4(or153)	10.1% (10/99)
	spd-1(oj5)	100% (83/83)
Comma-1.5 Fold	N2	100% (36/36)
	air-2(or207)	33.7% (31/92)
	zen-4(or153)	85.7% (54/63)
	spd-1(oj5)	100% (27/27)

Table S2. Quantification of DiI Staining of TS Mutants

Stage Before Shifting	Genotype	No Dil Signal	Weak Dil signal	Shape & Position Defect	Extended Dil Staining
15 °C Forever	N2	0% (0/32)	0% (0/32)	0% (0/32)	0% (0/32)
	air-2(or207)	2.7% (1/37)	8.1% (3/37)	2.7% (1/37)	0% (0/37)
	zen-4(or153)	0% (0/18)	0% (0/18)	0% (0/18)	0% (0/18)
	spd-1(oj5)	0% (0/34)	0% (0/34)	0% (0/34)	0% (0/34)
E4-E8	N2	0% (0/10)	0% (0/10)	0% (0/10)	0% (0/10)
	air-2(or207)	50% (1/2)	0% (0/2)	50% (1/2)	0% (0/2)
	zen-4(or153)	0% (0/0)	0% (0/0)	0% (0/0)	0% (0/0)
	spd-1(oj5)	0% (0/44)	4.5% (2/44)	9.1% (4/44)	6.8% (3/44)
E8-E16	N2	0% (0/29)	0% (0/29)	0% (0/29)	0% (0/29)
	air-2(or207)	16.7% (2/12)	58.3% (7/12)	58.3% (7/12)	0% (0/12)
	zen-4(or153)	77.8% (7/9)	11.1% (1/9)	22.2% (2/9)	0% (0/9)
	spd-1(oj5)	0% (0/59)	0% (0/59)	8.5% (5/59)	5.1% (3/59)
Comma-1.5 Fold	N2	0% (0/21)	0% (0/21)	0% (0/21)	0% (0/21)
	air-2(or207)	6.9% (2/29)	10.3% (3/29)	0% (0/29)	13.8% (4/29)
	zen-4(or153)	0% (0/53)	0% (0/53)	0% (0/53)	22.6% (12/53)
	spd-1(oj5)	0% (0/18)	0% (0/18)	0% (0/18)	33.3% (6/18)

Development • Supplementary information

Table S3. Strains used in this study.

Strain	Genotype	Origin
AZ212	*unc-119(ed3) iii; ruIs32[Ppi-1::GFP::His-58] iii*	(Praitis et al., 2001)
DKC21	*ltSi1016[pDC337; Pdyf-7::vhhGFP4::ZIF-1::dyf-7_3'UTR; cb-unc-119(+)] air-2(lt58[air-2::GFP::tev::loxP::3xFlag]) i;Pnphp-4::mNeonGreen-his-72:tbb-2_3'UTR;;gpd-2/3 operon linker-mKate2-PH:unc-34_3'UTR] v*	This Study
EKM48	*unc-119(ed3) iii; ojIs51[Ppie-1::GFP::air-2; unc-119(+)]*	(Bembenek et al., 2013)
EKM50	*unc-119(ed3) iii; ojIs51[Ppie-1::GFP::air-2; unc-119(+)]; ltIs37[(pAA64) pie-1p::mCherry::his-58 + unc-119(+)] iv; ltIs44[pie-1p::mCherry::PH(PLC1delta1) + unc-119(+)] v*	(Bembenek et al., 2013)
EKM51	*unc-119(ed3) iii; ojIs51[Ppie-1::GFP::air-2; unc-119(+)]; ltIs37[(pAA64) pie-1p::mCherry::his-58 + unc-119(+)] iv*	This Study
EKM52	*unc-119(ed3) iii; ojIs51[Ppie-1::GFP::air-2; unc-119(+)];ltIs44[pie-1p::mCherry::PH(PLC1delta1) + unc-119(+)] v*	This Study
EU630	*air-2(or207) i*	(Severson et al., 2000)
EU716	*zen-4(or153) iv*	(Severson et al., 2000)
JA1559	*unc-119(ed3) iii; weIs21[pJA138 (pie-1::mCherry::tub::pie-1)]*	(Lee et al., 2015)
JAB23	*unc-119(ed3) iii; ojIs51[Ppie-1::GFP::air-2; unc-119(+)]; weIs21[pJA138 (pie-1::mCherry::tub)]*	This Study

Development • Supplementary information

JAB24	*zen-4(or153ts) iv; xsEx6[zen-4::GFP; rol-6 (su1006)]; unc-119(ed3) iii; weIs21[pJA138 (pie-1::mCherry::tub)]*	This Study
JAB32	*unc-119(ed3) iii; ddIs26[pie-1p::mCherry::par-6; unc-119(+)] v; ojIs51[Ppie-1::GFP::air-2; unc-119(+)]*	This Study
JAB34	*unc-119(ed3) iii; zen-4(or153) iv; xsEx6[zen-4::GFP; rol-6 (su1006)]; ltIs44[pie-1p::mCherry::PH(PLC1delta1) + unc-119(+)] v*	This Study
JAB36	*unc-119(ed3) iii; ddIs26[pie-1p::mCherry::par-6; unc-119(+)] v; ojIs51 [Ppie-1::GFP::air-2 + unc-119(+)]*	This Study
JAB38	*unc-119(ed3) iii; air-2(or207); ltIs44[pie-1p::mCherry::PH(PLC1delta1) + unc-119(+)] v; ojIs37[Ppie-1::H2B::mCherry; unc-119(+)] iv; ojIs2[alpha-tubulin::GFP unc-119(+)]*	This Study
JAB39	*unc-119(ed3) III; ojIs51[Ppie-1::GFP::air-2 + unc-119(+)]; ruIs32[Ppi-1::GFP::His-58; unc-119(ed3) iii; ddIs6[tbg-1::GFP + unc-119(+)] v*	This Study
JAB52	*unc-119(ed3) iii; ddIs6[tbg-1::GFP + unc-119(+)] v; ruIs32[Ppi-1::GFP::His-58; unc-119(ed3) iii; weIs21[pJA138 (pie-1::mCherry::tub)]*	This Study
JAB60	*unc-119(ed3) iii; ojIs51[Ppie-1::GFP::air-2; unc-119(+)]; pwIs476[Ppie-1::mCherry::rab-11]*	This Study
JAB116	*unc-119(ed3) iii; weIs21[pJA138 (Ppie-1::mCherry::tub)]; unc-119(+)]; zuIs45[nmy-*	This Study

Development • Supplementary information

	2::NMY-2::GFP; unc-119(+)] v	
JAB142	*unc-119(ed3) iii; ojIs2[alpha-tubulin::GFP unc-119(+)]; ltIs37[(pAA64) pie-1p::mCherry::his-58 + unc-119(+)] iv; ltIs44[pie-1p::mCherry::PH(PLC1delta1) + unc-119(+)] v*	This Study
JAB194	*air-2(erb80[air-2::mScarlet]) i*	This Study
JAB200	*hmp-1(cp20[hmp-1::gfp + LoxP unc-119(+) LoxP]) v; weIs21[pJA138 (pie-1::mCherry::tub::pie-1)]*	This Study
JAB205	*air-2(erb-81[air-2::linker::GFP])*	This Study
JAB207	*unc-119(ed3) iii; air-2(or207) i; hmp-1(cp20[hmp-1::gfp + LoxP unc-119(+) LoxP]) v; ltIs44[pie-1p::mCherry::PH(PLC1delta1) + unc-119(+)] v; ojIs37[Ppie-1::H2B::mCherry; unc-119(+)] iv*	This Study
JAB210	*air-2(erb80[air-2::mScarlet) i; zuIs45[nmy-2::NMY-2::GFP; unc-119(+)] v*	This Study
JAB223	*unc-119(ed3) iii; air-2(erb-81[air-2::linker::GFP]); ltSi910[pOD2044/pSW378; Pelt-2::vhhGFP4::ZIF-1::operon-linker::mCherry::histone::tbb-2_3'UTR; cb-unc-119(+)] ii*	This Study
JAB224	*unc-119(ed3) iii; zen-4(lt30[GFP::loxP::zen-4]) iv; ltSi910[pOD2044/pSW378; Pelt-2::vhhGFP4::ZIF-1::operon-linker::mCherry::histone::tbb-2_3'UTR; cb-unc-119(+)] ii*	This Study
JAB225	*unc-119(ed3) iii; nmy-2(cp13[nmy-2::GFP + LoxP]) i; ltSi910[pOD2044/pSW378; Pelt-*	This Study

Development • Supplementary information

	2::vhhGFP4::ZIF-1::operon-linker::mCherry::histone::tbb-2_3'UTR; cb-unc-119(+)] ii	
JAB235	*spd-1(oj5) I; air-2(erb-81[air-2::linker::GFP]); ltIs37[(pAA64) pie-1p::mCherry::his-58 + unc-119(+)] iv; ltIs44[pie-1p::mCherry::PH(PLC1delta1) + unc-119(+)] v*	This Study
JCC401	*icp-1(or663ts) i*	(Davies et al., 2014)
JJ1473	*unc119(ed3) iii; zuIs45[nmy-2p::nmy-2::GFP; unc-119(+)] v*	(Nance et al., 2003)
LP162	*nmy-2(cp12[nmy-2::GFP + LoxP] i*	(Dickinson et al., 2013)
LP169	*unc-119(ed3) iii; hmp-1(cp21[hmp-1::GFP _ LoxP unc-119(+) LoxP]) v*	(Marston et al., 2016)
MG170	*zen-4(or153) iv; xsEx6[zen-4::GFP + rol-6(su1006)*	(Kaitna et al., 2000)
N2	Bristol (wild-type)	CGC
OD56	*unc-119(ed3) iii; ltIs37[(pAA64) pie-1p::mCherry::his-58 + unc-119(+)] iv*	(McNally et al., 2006)
OD70	*unc-119(ed3) iii; ltIs44[pie-1p::mCherry::PH(PLC1delta1) + unc-119(+)] v*	(Kachur et al., 2008)
NWG002	*unc-119(ed3) iii; ltIs44[pie-1p::mCherry::PH(PLC1delta1) + unc-119(+)] v; zuIs45[nmy-2p::nmy-2::GFP; unc-119(+) v*	(Redemann et al., 2010)
OD2768	*unc-119(ed3) iii; ltSi910[pOD2044/pSW378; Pelt-2::vhhGFP4::ZIF-1::operon-linker::mCherry::histone::tbb-2_3'UTR; cb-unc-119(+)] ii*	(Wang et al., 2017)

Development • Supplementary information

OD2979	*zen-4(lt30[GFP::loxP::zen-4]) iv*	(Lee et al., 2018)
OD3025	*unc-119(ed3) iii; ltSi1016[pDC337; Pdyf-7::vhhGFP4::ZIF-1::dyf-7_3'UTR; cb-unc-119(+)] i #2*	(Cheerambathur et al., 2019)
OD3230	*air-2(lt58[air-2::GFP::tev::loxP::3xFlag]) i*	(Cheerambathur et al., 2019)
OD3262	*ltSi1016[pDC337; Pdyf-7::vhhGFP4::ZIF-1::dyf-7_3'UTR; cb-unc-119(+)] air-2(lt58[air-2::GFP::tev::loxP::3xFlag]) i*	(Cheerambathur et al., 2019)
OD3919	*unc-119(ed3) iii; ltSi1174[oxTi365; pDC591; Pnphp-4::mNeonGreen-his-72: tbb-2_3'UTR;;gpd-2/3 operon linker-mKate2-PH:unc-34_3'UTR] v*	(Cheerambathur et al., 2019)
RT1196	*unc-119(ed3) iii; pwIs476[Ppie-1:mCherry:RAB-11.1, unc-119(+)]*	Gift from Barth Grant
SA240	*unc-119(ed3) iii; tjIs54[pie-1 promoter-gfp::tbb-2; pie-1 promoter-2xmCherry::tgb-1; unc-119^{+}]*	(Toya et al., 2010)
SA245	*unc-119(ed3) iii; tjIs57[pie-1 promoter-mCherry::his-48; unc-119^{+}]*	(Toya et al., 2010)
SA250	*unc-119(ed3) iii; tjIs54[pie-1 promoter-gfp::tbb-2; pie-1 promoter-2xmCherry::tgb-1; unc-119^{+}]; tjls57[pie-1 promoter-mCherry::his-48; unc-119^{+}]*	(Toya et al., 2010)
TH27	*unc-119(ed3) iii; ddIs6[tbg-1::GFP + unc-119(+)] v*	(Redemann et al., 2010)
TH110	*unc-119(ed3) iii; ddIs26[pie-1p::mCherry::par-6; unc-119(+)]*	(Schonegg et al., 2007)
WH12	*spd-1(oj5) i*	(O'Connell et al., 1998)
WH210	*unc-119(ed3) iii; ojIs2[alpha-tubulin::GFP unc-119(+)]*	(Kemp et al., 2004)

Development • Supplementary information

Bibliography

Bembenek, J.N., Verbrugghe, K.J., Khanikar, J., Csankovszki, G., and Chan, R.C. (2013). Condensin and the spindle midzone prevent cytokinesis failure induced by chromatin bridges in C. elegans embryos. Curr Biol *23*, 937-946.
Cheerambathur, D.K., Prevo, B., Chow, T.L., Hattersley, N., Wang, S., Zhao, Z., Kim, T., Gerson-Gurwitz, A., Oegema, K., Green, R., *et al.* (2019). The Kinetochore-Microtubule Coupling Machinery Is Repurposed in Sensory Nervous System Morphogenesis. Dev Cell *48*, 864-872 e867.
Davies, T., Jordan, S.N., Chand, V., Sees, J.A., Laband, K., Carvalho, A.X., Shirasu-Hiza, M., Kovar, D.R., Dumont, J., and Canman, J.C. (2014). High-resolution temporal analysis reveals a functional timeline for the molecular regulation of cytokinesis. Dev Cell *30*, 209-223.
Dickinson, D.J., Ward, J.D., Reiner, D.J., and Goldstein, B. (2013). Engineering the Caenorhabditis elegans genome using Cas9-triggered homologous recombination. Nat Methods *10*, 1028-1034.
Kachur, T.M., Audhya, A., and Pilgrim, D.B. (2008). UNC-45 is required for NMY-2 contractile function in early embryonic polarity establishment and germline cellularization in C. elegans. Dev Biol *314*, 287-299.
Kaitna, S., Mendoza, M., Jantsch-Plunger, V., and Glotzer, M. (2000). Incenp and an aurora-like kinase form a complex essential for chromosome segregation and efficient completion of cytokinesis. Curr Biol *10*, 1172-1181.
Kemp, C.A., Kopish, K.R., Zipperlen, P., Ahringer, J., and O'Connell, K.F. (2004). Centrosome maturation and duplication in C. elegans require the coiled-coil protein SPD-2. Dev Cell *6*, 511-523.
Lee, K.Y., Esmaeili, B., Zealley, B., and Mishima, M. (2015). Direct interaction between centralspindlin and PRC1 reinforces mechanical resilience of the central spindle. Nat Commun *6*, 7290.
Lee, K.Y., Green, R.A., Gutierrez, E., Gomez-Cavazos, J.S., Kolotuev, I., Wang, S., Desai, A., Groisman, A., and Oegema, K. (2018). CYK-4 functions independently of its centralspindlin partner ZEN-4 to cellularize oocytes in germline syncytia. Elife *7*.
Marston, D.J., Higgins, C.D., Peters, K.A., Cupp, T.D., Dickinson, D.J., Pani, A.M., Moore, R.P., Cox, A.H., Kiehart, D.P., and Goldstein, B. (2016). MRCK-1 Drives Apical Constriction in C. elegans by Linking Developmental Patterning to Force Generation. Curr Biol *26*, 2079-2089.
McNally, K., Audhya, A., Oegema, K., and McNally, F.J. (2006). Katanin controls mitotic and meiotic spindle length. J Cell Biol *175*, 881-891.
Nance, J., Munro, E.M., and Priess, J.R. (2003). C. elegans PAR-3 and PAR-6 are required for apicobasal asymmetries associated with cell adhesion and gastrulation. Development *130*, 5339-5350.
O'Connell, K.F., Leys, C.M., and White, J.G. (1998). A genetic screen for temperature-sensitive cell-division mutants of Caenorhabditis elegans. Genetics *149*, 1303-1321.
Praitis, V., Casey, E., Collar, D., and Austin, J. (2001). Creation of low-copy integrated transgenic lines in Caenorhabditis elegans. Genetics *157*, 1217-1226.
Redemann, S., Pecreaux, J., Goehring, N.W., Khairy, K., Stelzer, E.H., Hyman, A.A., and Howard, J. (2010). Membrane invaginations reveal cortical sites that pull on mitotic spindles in one-cell C. elegans embryos. PLoS One *5*, e12301.
Schonegg, S., Constantinescu, A.T., Hoege, C., and Hyman, A.A. (2007). The Rho GTPase-activating proteins RGA-3 and RGA-4 are required to set the initial size of PAR domains in Caenorhabditis elegans one-cell embryos. Proc Natl Acad Sci U S A *104*, 14976-14981.
Severson, A.F., Hamill, D.R., Carter, J.C., Schumacher, J., and Bowerman, B. (2000). The aurora-related kinase AIR-2 recruits ZEN-4/CeMKLP1 to the mitotic spindle at metaphase and is required for cytokinesis. Curr Biol *10*, 1162-1171.
Toya, M., Iida, Y., and Sugimoto, A. (2010). Imaging of mitotic spindle dynamics in Caenorhabditis elegans embryos. Methods Cell Biol *97*, 359-372.
Wang, S., Tang, N.H., Lara-Gonzalez, P., Zhao, Z., Cheerambathur, D.K., Prevo, B., Chisholm, A.D., Desai, A., and Oegema, K. (2017). A toolkit for GFP-mediated tissue-specific protein degradation in C. elegans. Development *144*, 2694-2701.
